# Valorization of Quince By-Products Using Natural Deep Eutectic Solvents (NADES): Extraction and In Vitro Digestion of Phenolic Compounds

**DOI:** 10.3390/foods14203507

**Published:** 2025-10-15

**Authors:** Erturk Bekar

**Affiliations:** Department of Food Engineering, Faculty of Agriculture, Bursa Uludag University, Gorukle, 16059 Bursa, Türkiye; erturk@uludag.edu.tr

**Keywords:** *Cydonia oblonga*, phenolic bioaccessibility, green extraction, choline chloride, phenolic profile, antioxidant capacity

## Abstract

Quince (*Cydonia oblonga* Mill.) processing generates peel and core by-product fractions that are underexploited resources with untapped potential for valorization in sustainable food systems. In this study, ultrasound-assisted extraction was performed using several choline chloride-based natural deep eutectic solvents (NADES, six formulations with distinct hydrogen-bond donors) and compared with 70% (*v*/*v*) ethanol. Extracts were analyzed for total phenolic content, antioxidant capacity, and individual phenolic compounds by LC-MS/MS, and their bioaccessibility was determined through a standardized in vitro digestion model. Organic acid-based NADES, particularly ChCl:MA (2:1) and ChCl:LA (1:1), yielded significantly higher phenolic contents from the peel than ethanol (up to ~45% increase, *p* < 0.05), and ChCl:MA maintained superior antioxidant capacity after digestion. In the core fraction, glucose- and glycerol-based NADES promoted the release of bound phenolics, resulting in bioaccessibility values exceeding 100%, indicating the release of previously bound phenolics under digestive conditions. The present study provides novel insights into the effects of NADES on both extraction efficiency and digestibility of quince by-products. These findings highlight quince peel and core as promising raw materials for developing functional food and nutraceutical ingredients, thereby offering a feasible strategy for upcycling fruit-processing residues into health-promoting applications.

## 1. Introduction

The food sector is constantly growing to meet the increasing demands of the global population, and the fruit and vegetable processing industry also contributes to this growth. The proportion of waste and by-products produced by fruits and vegetables processed in these businesses is reported to be 25–30%, varying from product to product. The circular bioeconomy, which focuses on the sustainable use of biological resources, positions these products as valuable raw materials for the recovery of bioactive compounds [1,2,3,4,5]. This approach aligns with the green extraction framework and promotes polyphenol recovery using low-hazard solvents [2,6].

In this study, peel and core fractions were prepared as laboratory-simulated residues rather than industrial by-products, in order to minimize the confounding effects of enzymatic browning, microbial activity, and oxidative changes that are typical of industrial waste streams. This approach, commonly used in fruit by-product valorization research, ensures reproducibility and allows solvent-related effects to be isolated more clearly [7,8]. Quince (*Cydonia oblonga* Mill.), introduced in the following section, was selected as the model fruit for this framework.

Quince belongs to the Maloideae subfamily of the Rosaceae family. Türkiye is the world’s leading producer of quince (192,237 tons in 2023), while global production exceeds 798 thousand tons [9,10]. Traditionally used in the production of products such as jam, marmalade, and fruit juice, quince is a firm-textured fruit that is also consumed fresh. Industrial quince processing generates peels and cores/seeds as by-products (~10% by mass), which is lower than in many fruits yet still substantial at production scale [11,12,13]. Classic chemical profiling studies have shown that quince peel, which stands out among these by-products, has a significantly higher phenolic content compared to pulp [14,15]. Additionally, extracts obtained from quince by-products, particularly quince peel, have been demonstrated to have antioxidant, antimicrobial, and anti-inflammatory effects. This makes quince attractive as a functional food ingredient [11,16,17].

Previous studies show that quince peel extracts are phenolic-rich and exhibit measurable in vitro antioxidant activity, supporting their use as functional ingredients [18,19]. At the same time, in industrial quince processing, peel and core fractions are frequently diverted to low-value uses (e.g., feed, compost) or discarded, indicating limited valorization in many settings [20], whereas recent assessments support the potential of quince by-products as functional ingredients owing to their composition and antioxidant capacity [21].

Organic solvents have traditionally been used for polyphenol recovery. However, environmental and safety limitations persist for these solvents [22]. Therefore, natural deep eutectic solvents (NADES), composed of bio-compatible components (e.g., choline chloride + organic acid/sugar), are emerging as biodegradable, adaptable, and highly effective extraction alternatives [2,6,23]. The type of hydrogen donor component (HBD) and water content of NADES have been shown to significantly affect phenolic solubility by adjusting the viscosity and polarity of the solvent. It is also reported to offer favorable antimicrobial/antioxidant profiles [23,24,25]. Consistent with this rationale, choline chloride-based NADES, with viscosity adjusted by water, have increased phenolic extraction and supported extract stability in orange peel [26,27], and improved polyphenol recovery and extraction kinetics in cocoa bean shells relative to conventional media [25].

Low-temperature conditions during phenolic extraction are critical for preventing their degradation. Ultrasound-assisted extraction is often used to accelerate the extraction process and obtain high yields. When combined with NADES, the process achieves high yields at lower temperatures and in shorter times, reflecting a more favorable environmental profile [28,29]. Consistent with this, choline chloride-based NADES optimized via viscosity modulation by water markedly increase total phenolic content (TPC) and total antioxidant capacity (TAC) in polyphenol extraction from plant peels [25,30]. In a study conducted on walnut shells from walnut by-products, solvent–matrix interaction was examined from a bioaccessibility perspective, and it was reported that phenolics were efficiently extracted with ultrasound-assisted extraction (UAE) + NADES, and the resulting fractions were examined within the in vitro bioaccessibility window [31].

While studies conducted explicitly on quince indicate that it is rich in the phenolic profile and bioactivity of traditional organic solvent extracts [12,14,15], data on the post-digestion bioaccessibility of extracts obtained from quince peel and core using NADES have not been investigated. However, the decisive factor for consumer health is the release of extract components during digestion and their transition to the soluble phase, i.e., their bioaccessibility; a high extract content alone is not sufficient [31]. Furthermore, NADES formulation (hydrogen-bond acceptor (HBA)/HBD selection and water percentage) and UAE application may have different effects on the persistence of individual phenolics and their interactions in the matrix [23,25]. The present study addresses this limitation by (i) comparing polyphenol recovery from quince peel (QP) and quince core (QC, seed included inner fraction) by UAE with conventional ethanol and NADES, (ii) determination of individual phenolic content by LC-MS/MS system, (iii) evaluation of total and individual bioaccessibility of phenolic compounds after static in vitro digestion model. Thus, it aims to provide a feasible framework for translating the green solvent–process combination into the development of functional components from quince by-products.

## 2. Materials and Methods

### 2.1. Materials and Sample Preparation

Quince fruits were supplied from the local market. Peel (QP) and core (QC) were prepared as laboratory-simulated by-products. The washed quinces were peeled, the fruit was cut open, and the core was removed. Peels were removed manually with a stainless-steel knife to a target thickness of approximately 1–1.5 mm. Both the QP and the QC were dried in a freeze-dryer (INOFD-12PUP, Innova Bio-Meditech Co., Ltd., Qingdao, China) at −86 °C for 40 h, under a vacuum range of 10–20 Pa, making them ready for grinding. The dried samples were pulverized using an analytical grinder (A11 basic, IKA^®^-Werke GmbH & Co. KG, Staufen, Germany). Powders were passed through a 250 µm stainless-steel sieve (200 × 50 mm, 60 mesh, ASTM E11; Retsch GmbH, Haan, Germany) to standardize particle size (<250 µm) and then stored at −20 °C until analysis. All experimental parameters are reported in SI units, and centrifugal forces are expressed as relative g-force (×*g*) rather than revolutions per minute (rpm) to ensure reproducibility across instruments. Standardizing peel thickness and particle size in this manner minimizes variability in extraction efficiency associated with diffusion path length and surface area, as also emphasized in previous studies on fruit by-products [7,32].

### 2.2. Preparation of Choline Chloride-Based NADES

In preparing the NADES, choline chloride (ChCl, ≥98%; Sigma-Aldrich, Steinheim, Germany) was used as the hydrogen-bond acceptor. The hydrogen-bond donors were lactic acid (LA, >85%; Sigma-Aldrich, Steinheim, Germany), malic acid (MA, >99.0%; TCI, Tokyo, Japan), citric acid (CA, >98.0%; TCI, Tokyo, Japan), glucose (Glu, >98.0%; Merck, Darmstadt, Germany), glycerol (Gly, >99.0%; Merck, Darmstadt, Germany), and xylitol (Xyl > 98.5%; Kimyalab, Istanbul, Turkey). NADES mixtures were prepared at the molar ratios reported in previous studies and mixed at 80 °C in a shaking water bath (Nuve ST 30, Ankara, Turkey) until homogeneous, colorless liquids formed. Distilled water (30%, *v*/*v*) was then added to lower viscosity; the mixtures were vortexed for 60 s and treated in an ultrasonic bath (Isolab, Wertheim, Germany) for 5 min. The 30% water addition was intended to enhance mass transfer and improve interactions with plant matrices by reducing inherent viscosity. This level was chosen based on reports that 30% water can notably improve extraction performance without disrupting the eutectic structure or hydrogen-bonding capacity of NADES [33,34]. Maintaining this balance is critical: too much water weakens the unique solvent properties of NADES, whereas too little leaves them highly viscous and less practical to use [34,35]. A 70% ethanol solution was used for comparison (Table 1). Ethanol (70%) is commonly used as a benchmark for extracting plant phenolics because hydroethanolic mixtures in the ~30–70% *v*/*v* range balance polarity and mass transfer; accordingly, many studies and reviews adopt ~70% ethanol as a standard condition [36,37,38].

### 2.3. Characterization of NADES

#### 2.3.1. Density

One-milliliter aliquots of each NADES were weighed at room temperature with a semi-micro balance (AUW220D, Shimadzu, Tokyo, Japan).

The density was then calculated using the formula: *ρ* = *m*_NADES_/*V*_NADES_.

#### 2.3.2. Fourier Transform İnfrared (FTIR) Spectroscopy

FTIR spectra of NADES were measured at room temperature (Shimadzu IRTracer-100, Tokyo, Japan) in the wavelength range of 4000 to 500 cm^−1^, with a resolution of 4 cm^−1^ and 32 scans per spectrum.

### 2.4. Ultrasound-Assisted Extraction (UAE) of Phenolic Compounds

Extraction of phenolic compounds was performed as previously described in the literature [39]. As indicated in Table 1, 10 mL aliquots of NADES solutions or ethanol (70%) were added to each of the prepared QP and QC powders (0.5 g). Extractions were conducted in 50 mL conical tubes (10 mL working volume), capped, and held upright in the bath; the water level was adjusted to match the liquid level in the tubes. The mixture was placed in an ultrasonic bath (Isolab, Eschau, Germany) at 60 °C, 40 kHz, and 180 W for 30 min. The mixtures were then centrifuged at 11,180× *g* (Hitachi CF15RN, Tokyo, Japan) for 10 min, and supernatants were transferred to clean tubes. All extracts obtained were stored at 4 °C until analysis.

### 2.5. Total Phenolic Content

The TPC of the samples was determined as previously described [40]. Results were obtained using a gallic acid standard curve (prepared in distilled water immediately before use, concentration range: 10–400 mg L^−1^; R^2^ > 0.999) and reported as milligrams of gallic acid equivalent (mg GAE) per gram of dry weight (mg GAE g^−1^ dw). For each measurement, 100 μL of extract was added into a cuvette, followed by 750 μL of Folin–Ciocalteu reagent. After 5 min of incubation at room temperature, 750 μL of 6% (*w*/*v*) Na_2_CO_3_ solution was added, and the mixture was incubated for 90 min in the dark. To reduce matrix effects caused by NADES, given their reducing character, all mixtures were centrifuged at 11,180× *g* for 5 min before absorbance measurement. After this step, which helps to remove particulate matter and increase the accuracy of spectrophotometric readings, the mixtures were transferred to cuvettes and absorbance was measured at 725 nm in a spectrophotometer (Cary 60, Agilent Technologies, Santa Clara, CA, USA). Baseline absorbance was corrected against matched digestion blanks (gastric and intestinal) processed in parallel for each solvent system (70% ethanol or the corresponding NADES), i.e., each extract was read against its own digestion blank.

### 2.6. Total Antioxidant Capacity Assays (DPPH and CUPRAC)

The TAC of the samples was determined using 2,2-diphenyl-1-picrylhydrazyl (DPPH) and cupric ion-reducing antioxidant capacity (CUPRAC) assays [41,42].

DPPH assay was performed by mixing 100 µL of extract with 2.0 mL of DPPH working solution, incubating in the dark for 30 min at room temperature, and reading at 517 nm [41]. Trolox standards (10–200 mg L^−1^, prepared immediately before use) yielded R^2^ ≥ 0.999. Results were expressed as mg Trolox equivalents per g dry weight (mg TE g^−1^ dw).

CUPRAC assay was carried out by combining 100 µL of extract sequentially with 1.0 mL of Cu (II) chloride solution, 1.0 mL of neocuproine solution, 1.0 mL of ammonium acetate solution, and 1.0 mL of deionized water, which were incubated in the dark for 30 min at room temperature, and a reading was performed at 450 nm [42]. Trolox standards (100–600 mg L^−1^) provided R^2^ ≥ 0.996. DPPH and CUPRAC readings were blank-subtracted against solvent- and phase-matched digestion blanks (70% ethanol or the relevant NADES processed through the same gastric/intestinal step without sample), so that observed differences between solvents reflect extract composition rather than matrix effects.

### 2.7. Identification and Quantification of Phenolic Compounds Using UPLC-ESI-MS/MS

Phenolic compounds were identified and quantified using an ultra-performance liquid chromatography–electrospray ionization tandem mass spectrometry (UPLC-ESI-MS/MS) system (Shimadzu LC-MS/MS 8060, Kyoto, Japan). A C18 column (100 × 3 mm, 3 µm; GL Sciences, Tokyo, Japan) was used for analysis, and the MS was operated in both negative and positive ESI modes. Ultrapure water (Purelab Classic, ELGA LabWater, High Wycombe, UK) and acetonitrile (LC-MS Grade; Merck, Darmstadt, Germany) were used as mobile phases in a gradient elution (Table S1). Analyses were performed at a flow rate of 0.40 mL min^−1^, with the column temperature at 40 °C and the autosampler maintained at 4 °C; the injection volume was 10 µL [43]. The MRM transitions and MS parameters are summarized in Table 2, and the calibration/validation data (slope, intercept, R^2^, LOD, LOQ) are provided in Table 3.

Sample spectra were recorded in multiple reaction monitoring (MRM) mode, and data acquisition was performed using Shimadzu LabSolution software (version 5.91, Shimadzu, Kyoto, Japan). Compounds were quantified using the following external standards: 3,4-dimethoxycinnamic acid (3,4-DMCA), epicatechin ((-)-epicatechin), rutin, quercetin, kaempferol, and chlorogenic acid (3-caffeoylquinic acid) from TRC (Toronto Research Chemicals, Vaughan, ON, Canada); isoquercetin (quercetin-3-glucoside) and *p*-coumaric acid (4-hydroxycinnamic acid) were from Sigma-Aldrich. Standards were stored at −20 °C until preparation, in dry form, and their purity was ≥95–99%. The purity of methanol (Merck) used for stock solutions was LC-MS grade. Results are reported as µg g^−1^ (dw).

Target compounds were quantified by UPLC–ESI–MS/MS in MRM mode using compound-specific transitions and collision energies (Table 2). Quantification used external calibration with authentic standards; linearity across the working ranges and LOD/LOQ are reported in Table 3. Analyte identity was accepted when the retention time fell within the predefined window and the quantifier/qualifier ion ratio was within tolerance. Quantification relied on MRM selectivity; full baseline separation was not required when these criteria were met. Representative chromatograms are provided in Supplementary Figures S2 and S3.

### 2.8. In Vitro Digestion Simulation

The in vitro digestion model applied by Minekus et al. [46] was used in the study. Gastric and intestinal electrolyte solutions were freshly prepared in accordance with the INFOGEST protocol, including the relevant buffer salts and enzymes for each phase (e.g., NaCl, KCl, NaHCO_3_, and CaCl_2_; pepsin for the gastric step; pancreatin and bile salts for the intestinal step). Stock acid and base solutions (1 M HCl and 1 M NaOH) were prepared for pH adjustments as recommended in the protocol, with minor variations depending on the solvent systems.

Following gastric and intestinal digestions, 5 mL aliquots were taken from each sample. At each stage, aliquots were immediately adjusted with 0.1% of formic acid, centrifuged at 11,180× *g* for 10 min, and the clear supernatants were analyzed as the bioaccessible fractions (and stored at −20 °C until measurement). The remaining mixture proceeded to the next digestion step.

In parallel with the digestion experiments, blank digestions were performed for each solvent system (all NADES formulations and 70% ethanol) without the addition of enzymes and bile. In these blanks, the respective solvent was subjected to the same gastric and intestinal electrolyte solutions and incubation conditions as the test samples. Aliquots were collected from these blanks and processed under identical analytical procedures, and the resulting values were used as matched blanks in spectrophotometric assays. This approach allowed us to control for the intrinsic reducing properties of NADES and ethanol, as well as the potential contributions of the digestion medium itself, thereby ensuring that the reported TPC and TAC values reflect only the effects of the extracts [46].

Bioaccessibility (%) was calculated as the ratio of the compound concentration detected in the intestinal supernatant (post-digestion) to its initial concentration in the undigested extract, expressed as a percentage according to the following equation:

Bioaccessibility (%) = (C_intestinal supernatant_/C_undigested extract_) × 100

where C_intestinal supernatant_ is the concentration of the analyte quantified in the aqueous fraction of the intestinal digesta, and C_undigested extract_ represents the concentration of the same compound in the original undigested sample.

Finally, after the intestinal step, digesta were centrifuged under the same × *g* conditions, and the clear supernatants (intestinal micellar phase) were analyzed directly without further extraction, as recommended in static digestion protocols [46,47]. To ensure comparability with undigested extracts, matrix-matched blanks (electrolyte solutions without sample) and enzyme-free controls (electrolytes + bile, no enzymes) were included. These controls confirmed that the observed post-digest changes reflected sample responses rather than the background effects of the digestion media.

### 2.9. Statistical Analysis

Analyses were performed in triplicate, and results are expressed as the mean ± standard deviation. Data were statistically analyzed using SPSS software (version 25; Chicago, IL, USA). Differences between groups were evaluated using one-way analysis of variance followed by Tukey’s post hoc test, with *p* < 0.05 considered statistically significant, and were applied to TPC, TAC, and compound-level data reported in the tables; % bioaccessibility values were presented descriptively without post hoc comparisons. Prior to ANOVA, the assumptions of normality and homogeneity of variances were verified. Shapiro–Wilk’s test indicated that the data did not deviate from normal distribution (*p* > 0.05), and Levene’s test confirmed the homogeneity of variances (*p* > 0.05). These results supported the validity of applying parametric analysis (one-way ANOVA followed by Tukey’s post hoc test).

## 3. Results and Discussion

### 3.1. Physicochemical Properties of NADES

The densities of NADES were found to be in the range of 1.107–1.204 g cm^−3^ (Table 1). The highest density was recorded with ChCl:CA (2:1), 1.204 g cm^−3^, and the lowest with ChCl:LA (1:1), 1.107 g cm^−3^. This result indicates that the hydrogen-bonding agents used significantly affect the solvent properties.

The FTIR spectra of the synthesized NADES (Figure S1) show a broad O-H stretching band (3000–3500 cm^−1^) in all systems, consistent with extensive hydrogen-bonded hydroxyl networks typical of NADES. Acid-based mixtures (citric, lactic, malic) additionally exhibit a carbonyl region near 1700–1750 cm^−1^, whereas glucose- and glycerol-based systems lack a distinct C=O but display intense C-O/C-O-C modes in the 1200–1000 cm^−1^ fingerprint range. Together with aliphatic C-H stretching around 2950/2850 cm^−1^, these signatures support donor–acceptor interactions between choline chloride and the respective hydrogen-bond donors under the tested molar ratios (Figure S1).

### 3.2. Quince Peel

#### 3.2.1. Total Phenolic Content, Antioxidant Capacity, and Bioaccessibility (%)

In QP, TPC and TAC values showed significant differences (*p* < 0.05) depending on the solvent used and digestion phases (Table 4). In undigested extracts, the highest TPC values were obtained with ChCl:LA (1:1; 19.64 mg GAE/g) and ChCl:MA (2:1; 19.39 mg GAE/g), and the fact that these solvents provided higher yields than 70% ethanol (13.34 mg GAE/g) indicates that organic acid-based NADES could be effective alternatives for phenolic extracts (*p* < 0.05). Relative to 70% ethanol, these correspond to ~47% (ChCl:LA 1:1) and ~45% (ChCl:MA 2:1) higher undigested TPC (Table 2). This result is considered to be potentially due to the facilitation of partial hydrolysis of pectic polysaccharides in the plant cell wall by NADES containing organic acid, owing to their low viscosity and high hydrogen-bonding capacity [44,48].

A general decrease was observed in TPC and TAC values throughout the digestion process. In the gastric phase, TPC decreased in all solvents, while ChCl:Gly (1:2) maintained a relatively higher value (10.88 mg GAE/g). In the intestinal phase, ChCl:CA (2:1; 5.63 mg GAE/g) and ChCl:MA (2:1; 4.71 mg GAE/g) sustained the highest TPC levels (*p* < 0.05). Compared with ethanol (2.13 mg GAE g^−1^), ChCl:MA (2:1) retained ~121% higher TPC at the intestinal endpoint (Table 2). This decrease can be attributed to the sensitivity of phenolics to pH changes during digestion and their susceptibility to reactions like oxidation, polymerization, and hydrolysis [46,47,49].

Antioxidant capacity results varied according to the method used. In the CUPRAC test, ChCl:Glu (2:1), ChCl:Gly (1:2), and ChCl:Xyl (1:1) (~62–67 mg TE/g) were prominent in the pre-digestion stage, whereas ChCl:MA (2:1; 38.25 mg TE/g) showed a significant superiority in the intestinal phase (*p* < 0.05). At the intestinal endpoint, ChCl:MA (2:1) delivered ~382% higher CUPRAC (38.25 vs. 7.94 mg TE g^−1^) and ~94% higher DPPH (43.69 vs. 22.54) than ethanol (Table 2). This is consistent with CUPRAC’s response to both hydrophilic and lipophilic antioxidants under pH ~7 conditions [42,50]. The intestinal phase of the DPPH analysis revealed that ChCl:MA (2:1; 43.69%) achieved the highest antioxidant activity, supporting its stronger radical scavenging potential [51].

**Figure 1:**
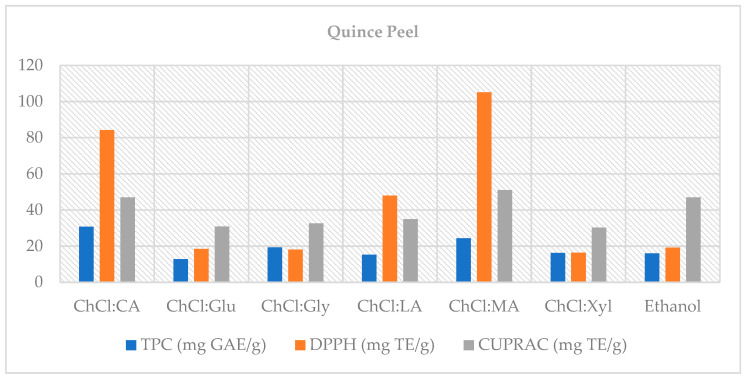
Bioaccessibility (%) of TPC and TAC (CUPRAC, DPPH) in QP extracts following in vitro digestion.

Bioaccessibility calculations more clearly revealed the differences between solvents after digestion (Figure 1). In QP, the highest TPC bioaccessibility (%) was found with ChCl:CA (2:1; 30.70%) and ChCl:MA (2:1; 24.29%), while CUPRAC bioaccessibility peaked with ChCl:MA (2:1; 51.03%), and DPPH bioaccessibility also peaked with ChCl:MA (2:1; 104.99%) (*p* < 0.05). The fact that DPPH bioaccessibility exceeded 100% is likely due to the release of bound phenolics during digestion [52] or the conversion of phenolics into derivatives with more potent antioxidant activity. For example, the conversion of flavonoid glycosides like rutin to the quercetin aglycone via hydrolysis is thought to lead to an increase in antioxidant activity [53]. Therefore, ChCl:MA (2:1) emerged as the most advantageous solvent in terms of post-digestion antioxidant capacity preservation.

The results revealed that organic acid-based NADES (especially ChCl:MA (2:1)) provide high yields in undigested extraction and offer a distinct advantage in the preservation and even enhancement of antioxidant capacity after digestion (*p* < 0.05). These findings confirm that the HBA/HBD type, ratio, and water content in the design of NADES can play a critical role in extraction efficiency and bioaccessibility [54].

Across solvents, the divergent trends in TPC, CUPRAC, and DPPH are consistent with the HBD identity, intrinsic acidity, polarity, and micro-viscosity of the NADES controlling both the extraction selectivity and post-digestion stability of phenolics. Acid-based NADES (e.g., ChCl:MA; ChCl:CA) are intrinsically acidic and form extensive H-bond networks with phenolic acids, which can stabilize labile species during the gastric step and facilitate their release from the plant matrix. In contrast, polyol-based systems (ChCl:Glu, ChCl:Gly, ChCl:Xyl) are near neutral yet more hydrophilic, often co-extracting sugars and other reducing agents that shape spectrophotometric outcomes. Water addition (30% *v*/*v*) reduces viscosity and enhances mass transfer without disrupting the eutectic structure, further modulating selectivity. These solvent-structure effects agree with our data, showing that ChCl:MA (2:1) outperforms in intestinal DPPH and CUPRAC, while ChCl:CA/ChCl:MA better preserved TPC after digestion [17,55,56,57].

Differences also arise from assay chemistry. TPC reflects total reducing capacity and is responsive to non-phenolic reducers; CUPRAC operates near neutral pH and captures a broad range of hydrophilic/lipophilic antioxidants; DPPH relies on a lipophilic radical and is particularly sensitive to solvent and pH. Consequently, solvent-driven changes in co-extracted acids, sugars, and glycosides—and their transformations during digestion—can shift the rank order across TPC, CUPRAC, and DPPH despite analyzing the same extracts. This framework explains why ChCl:MA yielded the highest intestinal DPPH, while acid-based NADES retained higher TPC values [45,50,58,59,60].

Finally, reaction mechanism, medium, kinetics/stoichiometry, and analyte coverage differ among methods: DPPH is dominated by fast, lipophilic scavengers; TPC integrates all reducers without weighting their efficiency; and LC–MS/MS quantifies only targeted analytes, overlooking newly formed or co-extracted antioxidants. Modest qualitative changes in composition can therefore reorder spectrophotometric outcomes without contradicting chromatographic totals [59,61]. A detailed compound-level interpretation follows in Section 3.2.2.

#### 3.2.2. Phenolic Acids and Bioaccessibility (%)

The pronounced pH sensitivity across solvents can be rationalized by the chemical stability of the dominant phenolic classes. Chlorogenic acids (caffeoylquinic acids) are prone to hydrolysis and isomerization during the gastric-to-intestinal transition, with larger losses around neutral pH; such transformations reduce intestinal recoveries and generate simpler phenolics (e.g., caffeic, quinic acids). By contrast, common flavonol glycosides (e.g., rutin, isoquercitrin) generally persist better during simulated digestion or are released from conjugated/bound forms, although their aglycones (e.g., quercetin) can be less stable at higher pH. These class-dependent behaviors agree with digestion studies tracking phenolic degradation under neutral/alkaline conditions [62,63,64,65].

Accordingly, the response to pH reflects extract composition. Acid-based NADES-enriched phenolic acids at extraction and showed sharper declines in TPC and caffeoylquinic acids after the intestinal step. In contrast, polyol-based NADES yielded a higher share of flavonol glycosides, which better preserved antioxidant readouts near neutral pH. INFOGEST-based digestions and studies following rutin/isoquercitrin or quercetin glucoside report similar patterns [66,67,68].

The LC–MS/MS operating conditions, MRM transitions, and calibration/validation parameters are detailed in Methods (Table 2 and Table 3). LC–MS/MS data were filtered according to the a priori criteria defined there (retention-time window and ion-ratio tolerances); features failing these criteria were not reported. Representative chromatograms are provided in Supplementary Figures S2 and S3.

In the QP fraction, chlorogenic acid was identified as the dominant phenolic compound (Table 5). This finding is consistent with the literature on the phenolic profile of quince; previous studies have also reported caffeoylquinic acids as the predominant phenolic class [9,14,69]. In the undigested stage, the highest chlorogenic acid value was measured in the 70% ethanol extract (192.61 µg/g), with ChCl:LA (1:1; 175.36 µg/g) and ChCl:Gly (1:2; 167.27 µg/g) presenting very close values. This indicates that hydroethanolic solvents provide effective phenolic release with low viscosity and a suitable polarity balance, but lactic acid and glycerol-based NADES can also be competitive alternatives with similar efficiency [54,70].

A sharp decrease was observed in all solvents during the gastric phase of digestion, but a partial recovery was recorded in the intestinal phase. For instance, the chlorogenic acid level increased from 16.48 µg/g to 20.97 µg/g with ChCl:Gly (1:2), from 13.06 µg/g to 21.92 µg/g with ChCl:LA (1:1), and from 14.58 µg/g to 24.08 µg/g with ethanol (70%) (*p* < 0.05). This pattern is consistent with the typical behavior described in the literature, where phenolic acids are lost through hydrolysis/isomerization under acidic conditions and partially recovered as solubility increases in the intestinal environment [46,47,71].

Among the minor phenolic acids, 3,4-dimethoxycinnamic acid and *p*-coumaric acid were detected at levels >LOQ only with ChCl:CA (2:1) and ChCl:MA (2:1) and decreased rapidly during digestion (*p* < 0.05). This suggests that organic acid-based NADES can exhibit selectivity towards specific phenolic subclasses and that the HBD structure determines phenolic solubility [23,25,27].

When evaluated in terms of total phenolic acids, the highest value in the undigested stage was obtained with ethanol (70%) (192.61 µg/g), while in the intestinal phase, ChCl:Gly (1:2; 20.97 µg/g) and ChCl:LA (1:1; 22.02 µg/g) solvents reached levels similar to ethanol (70%; 24.08 µg/g) (*p* < 0.05). This finding shows that NADES can provide a bioaccessible phenolic profile comparable to conventional solvents.

**Figure 2:**
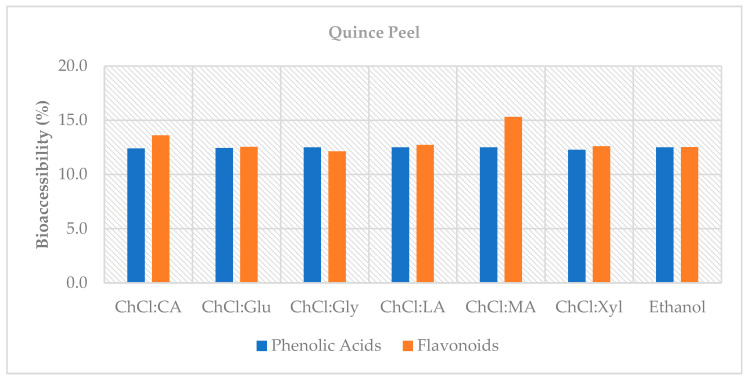
Bioaccessibility (%) of phenolic acids and flavonoids in QP extracts following in vitro digestion.

Bioaccessibility (%) values for QP phenolic acids remained constant at approximately 12% for all solvents (Figure 2). This reveals that the main factor limiting the measurable fraction after digestion is the structure of the food matrix and digestion conditions (pH changes, enzymatic hydrolysis, micellization with bile) rather than the solvent [31,39]. The advantage of NADES is not to increase the bioaccessibility percentage but to provide a similar absolute amount of phenolic acids as ethanol at the end of digestion by achieving high extraction yields with sustainable solvent systems. In sum, solvent choice not only shapes extraction yield but also the pH-dependent stability trajectory of phenolic acids versus flavonol glycosides during digestion.

#### 3.2.3. Flavonoids and Bioaccessibility (%)

The dominant flavonoids were identified as rutin and isoquercitrin in QP (Table 6). This finding is consistent with previous studies on the phenolic profile of quince [9,19,72]. In the undigested stage, the highest total flavonoid content was obtained with ethanol (70%, 209.91 µg/g) and ChCl:LA (1:1; 201.61 µg/g) (*p* < 0.05). For rutin, ChCl:LA (1:1; 124.26 µg/g) and ethanol (70%; 115.56 µg/g) were prominent, while for isoquercitrin, ChCl:LA (1:1; 71.55 µg/g) and ethanol (70%; 68.78 µg/g) stood out. For epicatechin, the highest values were obtained with ethanol (70%; 25.58 µg/g) and ChCl:Xyl (1:1; 21.04 µg/g). This pattern indicates that the compositions of NADES provide selective solubility for flavonol glycosides (rutin, isoquercitrin) and flavan-3-ols (epicatechin) due to their different polarity profiles [73,74].

The digestion simulation clearly revealed the stability and transformation processes of flavonoids. A significant decrease was observed for all flavonoids in the gastric phase, which can be explained by the tendency of glycosides, like rutin and isoquercitrin, to hydrolyze under low-pH conditions [75]. This hydrolysis led to the emergence of quercetin aglycone; indeed, in the ChCl:CA (2:1) and ChCl:MA (2:1) extracts, an increase in quercetin aglycone was observed in the gastric phase, while rutin and isoquercitrin decreased.

In the intestinal phase, the highest total flavonoid content was measured with ChCl:LA (1:1; 25.62 µg/g) and ethanol (70%; 26.26 µg/g); for isoquercitrin, these solvents again maintained the highest values (*p* < 0.05). This result shows that ChCl:LA (1:1) provides bioaccessible flavonoids at a level competitive with conventional solvents (Figure 2). Furthermore, in terms of bioaccessibility, ChCl:MA (2:1) stood out (15.29%), while other solvents and ethanol remained in the 12–13% range (*p* < 0.05). The dicarboxylic structure of malic acid may have provided this limited but significant advantage by supporting the solubility and stability of quercetin glycosides in the intestinal phase [27,76].

Epicatechin was the flavonoid that underwent the most rapid loss throughout digestion. This is consistent with the literature reporting that flavan-3-ols are unstable at neutral/alkaline pH; it has been reported that epicatechin and its derivatives can undergo rapid oxidative degradation within minutes under intestinal conditions [77,78,79].

### 3.3. Quince Core

#### 3.3.1. Total Phenolic Content, Antioxidant Capacity, and Bioaccessibility (%)

TPC and TAC profile of the QC fraction shows that this by-product is distinctly different from the QP (Table 7). In the undigested phase, the TPC values of QC were low, with the highest level measured in the ethanol extract (6.98 mg GAE/g). This indicates that the limited amount of free phenolics in QC can be easily dissolved with conventional solvents. However, it is known that a significant portion of phenolic compounds in QC exists in the form of non-extractable polyphenols (NEPPs) bound to cell-wall macromolecules [80].

The effect of the digestion process is a critical turning point for QC. While TPC and TAC values decreased with digestion in QP, an increase was observed in some solvents during digestion in QC, on the contrary. Particularly, the post-digestion increase in TPC in ChCl:CA (2:1), ChCl:Gly (1:2), and ChCl:MA (2:1) extracts (*p* < 0.05) suggests the release of bound phenolics as a result of enzymatic degradation and pH changes [46,47,81]. The loss of value in the ethanol extract during digestion is consistent with this solvent primarily dissolving free phenolics, which then degrade under digestion conditions. In contrast, NADES may have facilitated the release of bound phenolics during digestion by carrying the bound phenolic–matrix complexes, resulting in TPC levels that exceeded initial values.

**Figure 3:**
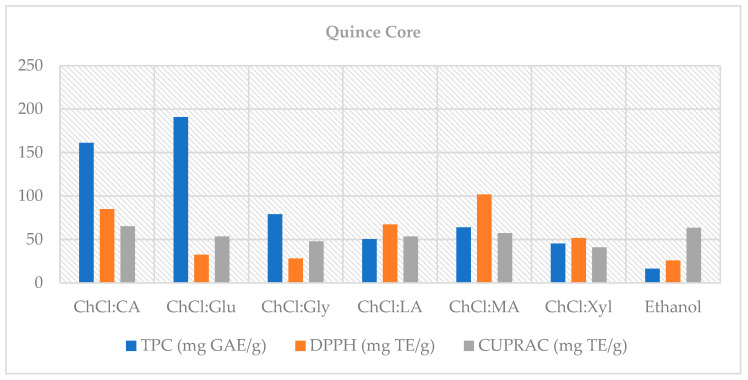
Bioaccessibility (%) of TPC and TAC (CUPRAC, DPPH) in QC extracts following in vitro digestion.

This was also reflected in the bioaccessibility calculations (Figure 3). TPC bioaccessibility (%) was found to be well over 100% for ChCl:Glu (2:1) (191%) and ChCl:CA (2:1) (161%). This does not indicate new phenolic formation, but rather the activation of initially unmeasurable bound fractions through digestion [52]. Thus, while glucose- and citric acid-based NADES promoted >100% of TPC bioaccessibility in QC, CUPRAC bioaccessibility remained comparable to ethanol (~57% vs. ~63%).

In terms of antioxidant capacity, CUPRAC results were determined to be high in the undigested phase with ChCl:Glu (2:1), ChCl:Gly (1:2), and ChCl:Xyl (1:1) ~31 mg TE/g), while ChCl:MA (2:1; 20.36 mg TE/g) stood out in the intestinal phase (*p* < 0.05). In the DPPH analysis, ChCl:MA (2:1; 26.31 mg TE/g) also gave the highest result in the intestinal phase (*p* < 0.05). This suggests that organic acid-based NADES can provide a more stable and bioaccessible antioxidant fraction post-digestion [42,51].

DPPH bioaccessibility was found to be ~102% for ChCl:MA (2:1), ~85% for ChCl:CA (2:1), and ~67% for ChCl:LA (1:1). CUPRAC bioaccessibility ranged from 40 to 65%, with ~57% for ChCl:MA (2:1) and ~63% for ethanol (70%). The fact that CUPRAC responds to both hydrophilic and lipophilic antioxidants around pH 7 and DPPH’s sensitivity to the lipophilic phase can explain the ranking differences between the methods [42,51].

In general, the digestion process in QC enabled the release of an initially hidden pool of bound phenolics; particularly, ChCl:CA (2:1)- and ChCl:MA (2:1)-based solvents yielded the strongest results in terms of TPC and TAC in the intestinal phase (*p* < 0.05). These findings suggest that organic acid-based NADES can be considered effective carrier systems not only in the extraction stage but also in post-digestion preservation and release processes [54,73,82].

#### 3.3.2. Phenolic Acids and Bioaccessibility (%)

In the QC fraction, phenolic acids were found at very low levels before digestion, with values remaining <LOQ in most solvents (Table 8). This can be attributed to phenolic compounds in QC, which exist mainly in a bound form as NEPPs. The covalent bonding of phenolics with cell-wall polysaccharides (cellulose, pectin, hemicellulose) and proteins results in the detection of low levels of free phenolics with standard extraction methods [81,83].

Digestion simulation clearly showed the release of these bound phenolics. Phenolic acid levels began to increase in the gastric phase and recorded a dramatic rise in the intestinal phase. For example, chlorogenic acid increased from 0.42 µg/g to 7.93 µg/g with ChCl:Glu (2:1), from 0.24 µg/g to 7.07 µg/g with ChCl:Gly (1:2), and from <LOQ to 5.76 µg/g with ethanol (*p* < 0.05). This finding may be attributed to the breakdown of the matrix structure by digestive enzymes and pH changes, which in turn releases the bound phenolics [47,52].

**Figure 4:**
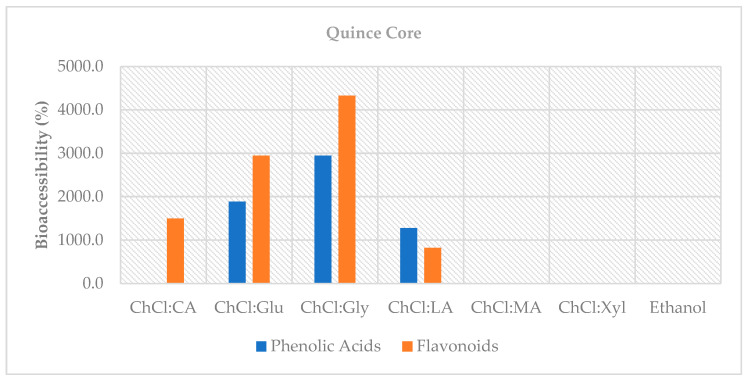
Bioaccessibility (%) of phenolic acids and flavonoids in QC extracts following in vitro digestion.

When bioaccessibility percentages were calculated, extremely high values such as ~1888% for ChCl:Glu (2:1), ~2946% for ChCl:Gly (1:2), and ~1281% for ChCl:LA (1:1) were found (Figure 4). This result does not reflect new phenolic formation, but rather the release of initially unmeasurable bound components through digestion. Therefore, the methodological limitations of bioaccessibility calculations in samples that are <LOQ in the undigested stage should be considered [46,47].

The ranking of results obtained post-digestion was ChCl:Glu (2:1) > ChCl:Gly (1:2) > ethanol > ChCl:LA (1:1). It has been reported that polyol (glycerol)- and saccharide (glucose)-based NADES increase the solubility of hydroxycinnamic acids (especially chlorogenic acid) through their dense hydrogen-bond networks and suitable viscosity/polarity profiles [23,27]. In contrast, the results in the intestinal phase were more limited for organic acid-based NADES (e.g., ChCl:LA (1:1)); this difference may highlight the effect of microenvironment pH and HBD properties on solubility/stability [74,82].

In general, although the QC fraction appeared poor in phenolic acids in the undigested stage (e.g., 3,4-DMCA, previously identified in quince [84] and also detected in the QP fraction in the present study), a significant amount of bioaccessible chlorogenic acid was released upon digestion (*p* < 0.05). This finding indicates that although QP data yielded higher results than QC, the core fraction can also be a valuable source of phenolics under digestive conditions [11,15]. Particularly, ChCl:Glu (2:1) and ChCl:Gly (1:2) emerged as the most successful solvents for chlorogenic acid release in the intestinal phase, presenting promising results for the evaluation of QC as a source of functional compounds.

#### 3.3.3. Flavonoids and Bioaccessibility (%)

In QC by-product, the vast majority of flavonoids remained at <LOQ levels in the undigested stage (Table 9). This can be attributed to the flavonoids being in a bound form in the matrix (NEPPs). These compounds, which are attached to polysaccharides and proteins by covalent bonds, may not be dissolved by standard extraction. Therefore, low initial values do not mean that QC is poor in flavonoids, but rather indicate the size of the bound fraction. This interpretation is consistent with the broader literature on NEPPs and their covalent association with cell-wall polymers, which become available primarily after gastrointestinal or colonic processes, and should be further quantified in future work to complete the QC profile [60,80].

The digestion simulation clearly showed the release of these bound flavonoids. While epicatechin was undetectable in the undigested stage, it became prominent in the intestinal phase with ChCl:Glu (2:1; 2.37 µg/g), ChCl:Gly (1:2; 2.38 µg/g), ChCl:Xyl (1:1; 1.61 µg/g), and ethanol (70%; 1.42 µg/g) (*p* < 0.05). This increase may be the result of the partial degradation of proanthocyanidin polymers during digestion, which would release monomeric epicatechin units [9,85].

Rutin and isoquercitrin were found at relatively high levels in the intestinal phase with ChCl:Glu (2:1) and ChCl:CA (2:1); furthermore, quercetin aglycone became apparent, especially with ChCl:MA (2:1; 0.77 µg/g) and ChCl:CA (2:1; 0.45 µg/g) (*p* < 0.05). This pattern is consistent with the partial hydrolysis of flavonol glycosides in a low-pH and enzymatic environment; the glycoside–aglycone conversion is considered the necessary first step for flavonoid bioaccessibility [75].

Total flavonoids in the intestinal phase were obtained at the highest levels with ChCl:Glu (2:1; 3.24 µg/g) and ChCl:Gly (1:2; 3.03 µg/g) (*p* < 0.05). This finding is consistent with the pattern in QC phenolic acids and suggests that glucose- and glycerol-based NADES facilitate enzymatic hydrolysis by hydrating the polysaccharide-rich matrix [31]. Ethanol lagged behind these solvents, indicating that NADES are superior in releasing bound fractions.

Bioaccessibility percentages were calculated to be extraordinarily high (Figure 4) in QC (e.g., ChCl:Glu (2:1) ~2945%, ChCl:Gly (1:2) ~4329%). This mathematical situation results from dividing the very low initial free values by the phenolics released from the bound pool during digestion. This indicates that QC is a significant reservoir of bound flavonoids released post-digestion.

The necessity of selecting an NADES formulation specific to the matrix is confirmed by the structural differences in the phenolic profiles and digestive behaviors of QP and QC. While NADES based on organic acids yielded the highest recovery of free phenolics from QP, the release of bound phenolics from QC during digestion was more effectively supported by NADES based on sugars and polyols. These findings indicate that the structural properties of the quince by-product and the form of the target phenolics (free vs. bound) are critical factors in selecting appropriate NADES for recovering functional ingredients.

Although choline chloride and the hydrogen-bond donors used here are food-compatible individually, their mixtures should be assessed as formulations for residual solvent levels, cytotoxicity, and sensory impact in the intended matrix. Future work should address recycling efficiency and regulatory compliance for residuals in the target applications.

## 4. Conclusions

This study demonstrates that choline chloride-based natural deep eutectic solvents (NADES)—particularly systems with organic-acid donors such as malic and lactic acid—offer efficient, lower-hazard alternatives to the conventional solvent (70% ethanol) for recovering phenolic compounds from quince by-products. In the peel, organic acid-based NADES (notably ChCl:MA 2:1 and ChCl:LA 1:1) achieved up to ~45% higher total phenolic content than ethanol and maintained superior antioxidant capacity after digestion; in the core fraction, glucose- and glycerol-based NADES exceeded 100% bioaccessibility, indicating the release of previously bound phenolics under digestive conditions and highlighting its potential as a functional source. Importantly, NADES enhanced extraction efficiency and helped retain antioxidant capacity after in vitro digestion, underscoring their value in valorizing quince by-products.

Nonetheless, the work is limited to a static in vitro digestion model, which cannot fully replicate the complexity of human gastrointestinal conditions. Moreover, the matrices were laboratory-simulated rather than factory-collected, and potential process-related differences were not evaluated. In addition, the study evaluated solely the soluble fraction, while insoluble bound phenolics were not considered, although their release during digestion could substantially contribute to the observed outcomes. Furthermore, only selected NADES formulations were assessed; the effects of solvent composition (including water content), solid-to-liquid ratio, temperature/time, and other process parameters warrant further optimization.

Future research should employ dynamic digestion systems, conduct in vivo validation, and advance pilot-scale trials to evaluate stability, safety, food-grade compliance, sensory and technological performance in real matrices, and solvent recyclability/reuse. In parallel, NADES should be assessed as mixtures, with measurement of residual solvent levels in the final product, basic cytotoxicity screening, and verification of compliance with applicable regulatory limits. From an application standpoint, the solvent components used here (choline chloride paired with organic acids or sugars) are commercially available in food-compatible grades. The feasibility of scale-up will depend primarily on viscosity control, the solid-to-liquid ratio, and operation at moderate temperatures. These parameters should be verified at pilot-scale together with solvent recovery and reuse. A focused techno-economic assessment is warranted to quantify costs and delineate the required unit operations.

Overall, the findings indicate that quince by-products are suitable raw materials and that ChCl-based NADES can be used to obtain phenolic ingredients for incorporation into food matrices. From a process point of view, choosing between ChCl-based NADES and 70% ethanol should be based on clear technical and economic factors: solvent cost and potential for reuse, viscosity and the energy needed to handle it, achievable extract concentration, and waste management. In this regard, large-scale adoption of NADES will likely depend on closed-loop solvent recovery/reuse and a demonstrable cost–benefit advantage over ethanol, as highlighted in techno-economic assessments of bio-based processes. To place the reported extraction gains in context, future work should therefore combine bioefficacy data with solvent-recycling metrics and life-cycle analysis.

## Figures and Tables

**Table 1:** Composition and density of choline chloride-based NADES containing 30% (*v*/*v*) H_2_O.

NADES	HBA	HBD	Molar Ratio	Density (g cm^−3^)
1	ChCl	CA	2:1	1.2042
2	ChCl	Glu	2:1	1.1696
3	ChCl	Gly	1:2	1.1543
4	ChCl	LA	1:1	1.1074
5	ChCl	MA	2:1	1.1438
6	ChCl	Xyl	1:1	1.1841

Abbreviations: ChCl, choline chloride; CA, citric acid; Glu, glucose; Gly, glycerol; LA, lactic acid; MA, malic acid; Xyl, xylitol. Formulations are given as molar ratios.

**Table 2:** Method parameters used for the identification of flavonoids and phenolic acids.

Compounds	Retention Time (RT)	Ionization Mode	Mass (*m*/*z*)	Main Fragment (*m*/*z*)	Other Fragmental Ions (*m*/*z*)	References
Flavonoids						
Epicatechin	2.44	ESI−	289.1	245.1	205.1	[7,44]
Rutin	3.10	ESI−	609.1	300.0	271.1	[12,13,44,45]
Isoquercitrin	3.45	ESI−	463.1	300.0	271.0	[7,13,15]
Quercetin	5.95	ESI−	301.0	151.2	179.0	[15,44]
Kaempferol	7.21	ESI−	285.0	117.1	151.0	[15]
Phenolic acids						
Chlorogenic acid	2.01	ESI−	353.1	191.1	110.9	[7,12,13,15,45]
*p*-Coumaric acid	3.54	ESI−	163.1	119.1	146.0; 117.1; 93.0; 65.0	[13,15]
3,4-DMCA	5.18	ESI+	208.8	191.0	163.1	[45]

**Table 3:** Calibration parameters, limits of detection (LODs), and quantification (LOQ) for the investigated phenolic compounds.

Phenolic Compound	Slope	Intercept	R^2^	LOD (µg/L)	LOQ (µg/L)
Flavonoids					
Epicatechin	51.776	18.015	0.995	0.092	0.306
Rutin	57.567	40.920	0.997	0.004	0.013
Isoquercitrin	245.081	491.437	0.995	0.005	0.015
Quercetin	218.947	624.068	0.995	0.001	0.004
Kaempferol	25.746	61.191	0.996	0.068	0.203
Phenolic acids					
Chlorogenic acid	221.011	364.509	0.996	0.063	0.189
*p*-Coumaric acid	108.059	265.154	0.995	0.054	0.161
3,4-DMCA	418.318	686.593	0.996	0.028	0.083

**Table 4:** Changes in TPC and TAC of QP extracts during in vitro digestion.

Solvent	Undigested	Gastric Digestion	Intestinal Digestion
TPC (mg GAE g^−1^)			
ChCl:CA	18.34 ± 1.15 ^ab,A^	9.58 ± 0.56 ^ab,B^	5.63 ± 0.49 ^a,C^
ChCl:Glu	14.95 ± 1.09 ^ab,A^	6.40 ± 0.31 ^c,B^	1.91 ± 0.05 ^b,C^
ChCl:Gly	13.50 ± 0.57 ^b,A^	10.88 ± 0.96 ^a,A^	2.61 ± 0.28 ^b,B^
ChCl:LA	19.64 ± 1.81 ^a,A^	9.97 ± 0.78 ^ab,B^	2.98 ± 0.26 ^b,C^
ChCl:MA	19.39 ± 1.89 ^a,A^	8.84 ± 0.58 ^abc,B^	4.71 ± 0.42 ^a,B^
ChCl:Xyl	16.25 ± 1.11 ^ab,A^	7.87 ± 0.73 ^bc,B^	2.63 ± 0.25 ^b,C^
Ethanol	13.34 ± 0.70 ^b,A^	9.88 ± 0.40 ^ab,B^	2.13 ± 0.12 ^b,C^
TAC (mg TE g^−1^)			
CUPRAC assay			
ChCl:CA	32.61 ± 2.61 ^b,A^	25.72 ± 0.57 ^ab,A^	27.45 ± 3.15 ^b,A^
ChCl:Glu	62.48 ± 2.71 ^a,A^	20.45 ± 1.13 ^b,B^	11.57 ± 1.23 ^d,C^
ChCl:Gly	67.24 ± 1.41 ^a,A^	21.58 ± 1.95 ^b,B^	12.20 ± 0.66 ^cd,C^
ChCl:LA	40.21 ± 3.47 ^b,A^	30.80 ± 1.76 ^a,A^	19.28 ± 1.57 ^c,B^
ChCl:MA	36.43 ± 1.03 ^b,A^	30.35 ± 2.87 ^a,A^	38.25 ± 3.26 ^a,A^
ChCl:Xyl	63.42 ± 4.59 ^a,A^	20.74 ± 1.20 ^bc,B^	10.42 ± 0.26 ^d,B^
Ethanol	41.26 ± 2.32 ^b,A^	14.22 ± 0.29 ^c,B^	7.94 ± 0.45 ^d,C^
DPPH assay			
ChCl:CA	82.13 ± 4.56 ^a,A^	74.08 ± 6.89 ^a,A^	38.53 ± 2.49 ^ab,B^
ChCl:Glu	79.29 ± 2.68 ^a,A^	70.08 ± 3.55 ^a,A^	24.52 ± 2.07 ^cd,B^
ChCl:Gly	81.09 ± 6.96 ^a,A^	70.36 ± 5.81 ^a,A^	26.41 ± 2.58 ^cd,B^
ChCl:LA	90.38 ± 2.44 ^a,A^	78.65 ± 6.08 ^a,A^	31.58 ± 1.01 ^bc,B^
ChCl:MA	85.62 ± 6.22 ^a,A^	80.07 ± 3.80 ^a,A^	43.69 ± 2.83 ^a,B^
ChCl:Xyl	76.16 ± 1.82 ^a,A^	65.91 ± 2.03 ^a,B^	23.01 ± 2.47 ^d,C^
Ethanol	48.05 ± 4.59 ^b,A^	35.37 ± 1.88 ^b,B^	22.54 ± 0.70 ^d,C^

ChCl:CA: ChCl:CA (2:1), ChCl:Glu: ChCl:Glu (2:1), ChCl:Gly: ChCl:Gly (1:2), ChCl:LA: ChCl:LA (1:1), ChCl:MA: ChCl:MA (2:1), ChCl:Xyl: ChCl:Xyl (1:1). Results are expressed as mean ± standard deviation. Different lower-case letters within the columns and upper-case letters within the rows represent statistically significant differences (*p* < 0.05). LOQ: limit of quantification.

**Table 5:** Phenolic acids in QP extracts during in vitro digestion by solvent (µg g^−1^).

Phenolic Acids	Solvent	Undigested	Gastric Digestion	Intestinal Digestion
3,4-DMCA	ChCl:CA	2.57 ± 0.06 ^a,A^	0.23 ± 0.004 ^a,B^	0.32 ± 0.009 ^a,B^
	ChCl:Glu	<LOQ	<LOQ	<LOQ
	ChCl:Gly	<LOQ	<LOQ	<LOQ
	ChCl:LA	0.77 ± 0.02 ^b,A^	0.07 ± 0.01 ^b,B^	0.10 ± 0.002 ^b,B^
	ChCl:MA	2.62 ± 0.10 ^a,A^	0.25 ± 0.005 ^a,B^	0.33 ± 0.008 ^a,B^
	ChCl:Xyl	<LOQ	<LOQ	<LOQ
	Ethanol	<LOQ	<LOQ	<LOQ
Chlorogenic acid	ChCl:CA	54.52 ± 3.83 ^d,A^	4.10 ± 0.35 ^d,B^	6.75 ± 0.32 ^d,B^
	ChCl:Glu	130.76 ± 8.27 ^c,A^	12.60 ± 0.34 ^b,B^	16.35 ± 1.39 ^c,B^
	ChCl:Gly	167.27 ± 14.13 ^ab,A^	16.48 ± 0.51 ^a,B^	20.97 ± 1.77 ^ab,B^
	ChCl:LA	175.36 ± 1.85 ^ab,A^	13.06 ± 1.15 ^b,C^	21.92 ± 0.23 ^ab,B^
	ChCl:MA	126.97 ± 6.22 ^c,A^	6.27 ± 0.29 ^c,B^	15.87 ± 0.85 ^c,B^
	ChCl:Xyl	150.99 ± 3.32 ^bc,A^	13.79 ± 0.02 ^b,B^	18.52 ± 0.92 ^bc,B^
	Ethanol	192.61 ± 1.07 ^a,A^	14.58 ± 0.23 ^ab,C^	24.08 ± 0.13 ^a,B^
*p*-Coumaric acid	ChCl:CA	1.47 ± 0.05 ^a^	<LOQ	0.18 ± 0.005 ^a^
	ChCl:Glu	0.81 ± 0.07 ^c^	<LOQ	<LOQ
	ChCl:Gly	<LOQ	<LOQ	<LOQ
	ChCl:LA	0.28 ± 0.03 ^d^	<LOQ	<LOQ
	ChCl:MA	1.23 ± 0.05 ^b^	<LOQ	0.16 ± 0.006 ^b^
	ChCl:Xyl	<LOQ	<LOQ	<LOQ
	Ethanol	<LOQ	<LOQ	<LOQ
∑Phenolic acids	ChCl:CA	58.57 ± 3.93 ^d,A^	4.32 ± 0.34 ^d,B^	7.26 ± 0.33 ^d,B^
	ChCl:Glu	131.57 ± 8.34 ^c,A^	12.60 ± 0.34 ^b,B^	16.35 ± 1.39 ^c,B^
	ChCl:Gly	167.77 ± 14.18 ^ab,A^	16.48 ± 0.51 ^a,B^	20.97 ± 1.77 ^ab,B^
	ChCl:LA	176.41 ± 1.86 ^ab,A^	13.13 ± 1.16 ^b,C^	22.02 ± 0.23 ^ab,B^
	ChCl:MA	130.82 ± 9.20 ^c,A^	6.52 ± 0.30 ^c,B^	16.35 ± 1.15 ^c,B^
	ChCl:Xyl	151.01 ± 3.32 ^bc,A^	13.79 ± 0.02 ^b,B^	18.52 ± 0.92 ^bc,B^
	Ethanol	192.61 ± 1.07 ^a,A^	14.58 ± 0.23 ^ab,C^	24.08 ± 0.13 ^a,B^

ChCl:CA: ChCl:CA (2:1), ChCl:Glu: ChCl:Glu (2:1), ChCl:Gly: ChCl:Gly (1:2), ChCl:LA: ChCl:LA (1:1), ChCl:MA: ChCl:MA (2:1), ChCl:Xyl: ChCl:Xyl (1:1). Results are expressed as mean ± standard deviation. Different lower-case letters within the columns and upper-case letters within the rows represent statistically significant differences (*p* < 0.05). LOQ: limit of quantification.

**Table 6:** Flavonoid profile of QP extracts during in vitro digestion by solvent (µg g^−1^).

Flavonoids	Solvent	Undigested	Gastric Digestion	Intestinal Digestion
Epicatechin	ChCl:CA	1.59 ± 0.09 ^d,A^	0.27 ± 0.006 ^c,B^	0.20 ± 0.009 ^e,B^
	ChCl:Glu	18.14 ± 0.92 ^b,A^	1.64 ± 0.12 ^b,B^	2.27 ± 0.12 ^b,B^
	ChCl:Gly	20.29 ± 1.32 ^b,A^	2.09 ± 0.18 ^a,B^	1.85 ± 0.15 ^c,B^
	ChCl:LA	4.91 ± 0.22 ^c,A^	0.25 ± 0.02 ^c,B^	0.61 ± 0.03 ^d,B^
	ChCl:MA	2.30 ± 0.07 ^cd,A^	0.09 ± 0.01 ^c,C^	0.29 ± 0.02 ^de,B^
	ChCl:Xyl	21.04 ± 0.19 ^b,A^	1.71 ± 0.07 ^b,C^	2.61 ± 0.05 ^b,B^
	Ethanol	25.58 ± 1.10 ^a,A^	2.31 ± 0.06 ^a,B^	3.20 ± 0.14 ^a,B^
Rutin	ChCl:CA	70.99 ± 3.68 ^d,A^	7.96 ± 0.16 ^cd,B^	8.87 ± 0.46 ^e,B^
	ChCl:Glu	71.03 ± 5.29 ^d,A^	10.45 ± 0.05 ^b,B^	8.88 ± 0.13 ^e,B^
	ChCl:Gly	103.48 ± 2.92 ^b,A^	11.47 ± 0.10 ^a,B^	12.94 ± 0.37 ^c,B^
	ChCl:LA	124.26 ± 1.37 ^a,A^	10.25 ± 0.05 ^b,C^	15.53 ± 0.17 ^a,B^
	ChCl:MA	69.98 ± 1.15 ^d^	7.49 ± 0.19 ^d^	8.81 ± 0.14 ^e^
	ChCl:Xyl	89.44 ± 2.68 ^c,A^	8.61 ± 0.24 ^c,B^	11.26 ± 0.22 ^d,B^
	Ethanol	115.56 ± 3.66 ^ab,A^	10.39 ± 0.42 ^b,C^	14.45 ± 0.07 ^b,B^
Isoquercitrin	ChCl:CA	36.32 ± 0.90 ^cd,A^	4.25 ± 0.05 ^d,B^	4.54 ± 0.11 ^d,B^
	ChCl:Glu	40.65 ± 3.85 ^c,A^	6.69 ± 0.05 ^ab,B^	5.08 ± 0.05 ^c,B^
	ChCl:Gly	54.44 ± 0.15 ^b,A^	7.20 ± 0.11 ^a,B^	6.81 ± 0.20 ^b,B^
	ChCl:LA	71.55 ± 0.68 ^a,A^	6.04 ± 0.03 ^b,C^	8.94 ± 0.11 ^a,B^
	ChCl:MA	32.44 ± 0.41 ^d,A^	3.32 ± 0.24 ^e,B^	4.06 ± 0.14 ^e,B^
	ChCl:Xyl	52.74 ± 1.74 ^b,A^	5.22 ± 0.27 ^c,B^	6.65 ± 0.15 ^b,B^
	Ethanol	68.78 ± 2.81 ^a,A^	6.33 ± 0.37 ^b,B^	8.60 ± 0.14 ^a,B^
Kaempferol	ChCl:CA	2.62 ± 0.11 ^b,A^	0.30 ± 0.04 ^b,B^	0.33 ± 0.01 ^b,B^
	ChCl:Glu	<LOQ	<LOQ	<LOQ
	ChCl:Gly	<LOQ	<LOQ	<LOQ
	ChCl:LA	0.90 ± 0.04 ^c^	<LOQ	<LOQ
	ChCl:MA	4.31 ± 0.13 ^a,A^	0.43 ± 0.008 ^a,B^	0.54 ± 0.02 ^a,B^
	ChCl:Xyl	<LOQ	<LOQ	<LOQ
	Ethanol	<LOQ	<LOQ	<LOQ
Quercetin	ChCl:CA	1.21 ± 0.03 ^B^	2.06 ± 0.12 ^b,A^	1.40 ± 0.02 ^b,B^
	ChCl:Glu	<LOQ	0.02 ± 0.001 ^d^	0.03 ± 0.004 ^d^
	ChCl:Gly	<LOQ	0.03 ± 0.001 ^d^	0.02 ± 0.005 ^d^
	ChCl:LA	<LOQ	0.33 ± 0.02 ^c^	0.53 ± 0.001 ^c^
	ChCl:MA	1.10 ± 0.10 ^C^	2.41 ± 0.05 ^a,B^	3.23 ± 0.06 ^a,A^
	ChCl:Xyl	<LOQ	0.01 ± 0.001 ^d^	0.02 ± 0.001 ^d^
	Ethanol	<LOQ	0.01 ± 0.002 ^d^	0.02 ± 0.001 ^d^
∑Flavonoids	ChCl:CA	112.73 ± 3.01 ^c,A^	14.84 ± 0.37 ^de,B^	15.34 ± 0.35 ^d,B^
	ChCl:Glu	129.83 ± 10.07 ^c,A^	18.80 ± 0.12 ^b,B^	16.26 ± 0.20 ^cd,B^
	ChCl:Gly	178.21 ± 4.11 ^b,A^	20.79 ± 0.03 ^a,B^	21.61 ± 0.50 ^b,B^
	ChCl:LA	201.61 ± 1.79 ^a,A^	16.86 ± 0.02 ^c,C^	25.62 ± 0.23 ^a,B^
	ChCl:MA	110.63 ± 1.40 ^c,A^	13.74 ± 0.50 ^e,B^	16.92 ± 0.06 ^c,B^
	ChCl:Xyl	163.21 ± 4.59 ^b,A^	15.55 ± 0.44 ^cd,B^	20.54 ± 0.41 ^b,B^
	Ethanol	209.91 ± 8.98 ^a,A^	19.03 ± 0.85 ^b,B^	26.26 ± 0.07 ^a,B^

ChCl:CA: ChCl:CA (2:1), ChCl:Glu: ChCl:Glu (2:1), ChCl:Gly: ChCl:Gly (1:2), ChCl:LA: ChCl:LA (1:1), ChCl:MA: ChCl:MA (2:1), ChCl:Xyl: ChCl:Xyl (1:1). Results are expressed as mean ± standard deviation. Different lower-case letters within the columns and upper-case letters within the rows represent statistically significant differences (*p* < 0.05). LOQ: limit of quantification.

**Table 7:** Changes in TPC and TAC of QC extracts during in vitro digestion.

Solvent	Undigested	Gastric Digestion	Intestinal Digestion
TPC (mg GAE g^−1^)			
ChCl:CA	1.83 ± 0.16 ^d,A^	1.87 ± 0.07 ^c,B^	2.95 ± 0.10 ^a,B^
ChCl:Glu	0.77 ± 0.08 ^e,A^	0.86 ± 0.04 ^d,B^	1.47 ± 0.15 ^bc,B^
ChCl:Gly	2.00 ± 0.18 ^d,A^	3.46 ± 0.24 ^b,B^	1.58 ± 0.18 ^bc,B^
ChCl:LA	3.68 ± 0.16 ^bc,A^	3.52 ± 0.19 ^b,A^	1.86 ± 0.12 ^b,B^
ChCl:MA	4.24 ± 0.05 ^b,A^	2.71 ± 0.15 ^bc,B^	2.71 ± 0.29 ^a,B^
ChCl:Xyl	3.40 ± 0.18 ^c,A^	1.90 ± 0.08 ^c,B^	1.54 ± 0.07 ^bc,B^
Ethanol	6.98 ± 0.38 ^a,A^	5.82 ± 0.53 ^a,A^	1.14 ± 0.09 ^c,B^
TAC (mg TE g^−1^)			
CUPRAC assay			
ChCl:CA	16.94 ± 1.15 ^b,A^	16.83 ± 1.39 ^b,A^	14.39 ± 0.79 ^b,A^
ChCl:Glu	31.08 ± 0.78 ^a,A^	11.43 ± 0.18 ^cd,B^	10.08 ± 1.16 ^c,B^
ChCl:Gly	31.75 ± 2.03 ^a,A^	12.33 ± 0.72 ^bcd,B^	8.91 ± 0.23 ^c,B^
ChCl:LA	20.03 ± 0.09 ^b,B^	23.10 ± 0.28 ^a,A^	13.47 ± 0.89 ^b,C^
ChCl:MA	20.02 ± 1.03 ^b,A^	23.02 ± 2.35 ^a,A^	20.36 ± 0.14 ^a,A^
ChCl:Xyl	31.44 ± 1.72 ^a,A^	13.49 ± 0.87 ^bc,B^	16.26 ± 0.76 ^b,B^
Ethanol	21.61 ± 0.67 ^b,A^	8.65 ± 0.61 ^d,B^	5.60 ± 0.53 ^d,C^
DPPH assay			
ChCl:CA	37.65 ± 2.22 ^b,A^	38.58 ± 2.99 ^a,A^	24.48 ± 2.67 ^ab,B^
ChCl:Glu	44.07 ± 1.37 ^ab,A^	39.53 ± 1.26 ^a,A^	23.54 ± 0.41 ^abc,B^
ChCl:Gly	45.01 ± 3.29 ^ab,A^	39.35 ± 0.63 ^a,A^	21.53 ± 0.10 ^bcd,B^
ChCl:LA	45.79 ± 3.41 ^ab,A^	43.57 ± 2.14 ^a,A^	24.55 ± 0.98 ^ab,B^
ChCl:MA	46.03 ± 1.18 ^ab,A^	44.35 ± 2.46 ^a,A^	26.31 ± 0.22 ^a,B^
ChCl:Xyl	47.97 ± 2.34 ^a,A^	39.66 ± 0.78 ^a,B^	19.62 ± 0.53 ^cd,C^
Ethanol	26.89 ± 0.50 ^c,A^	24.23 ± 0.19 ^b,A^	17.02 ± 1.11 ^d,B^

ChCl:CA: ChCl:CA (2:1), ChCl:Glu: ChCl:Glu (2:1), ChCl:Gly: ChCl:Gly (1:2), ChCl:LA: ChCl:LA (1:1), ChCl:MA: ChCl:MA (2:1), ChCl:Xyl: ChCl:Xyl (1:1). Results are expressed as mean ± standard deviation. Different lower-case letters within the columns and upper-case letters within the rows represent statistically significant differences (*p* < 0.05). LOQ: limit of quantification.

**Table 8:** Phenolic acids in QC extracts during in vitro digestion by solvent (µg g^−1^).

Phenolic Acids	Solvent	Undigested	Gastric Digestion	Intestinal Digestion
Chlorogenic acid	ChCl:CA	<LOQ	0.78 ± 0.03 ^c^	2.01 ± 0.06 ^e^
	ChCl:Glu	0.42 ± 0.02 ^a,C^	2.66 ± 0.08 ^a,B^	7.93 ± 0.23 ^a,A^
	ChCl:Gly	0.24 ± 0.02 ^b,B^	2.38 ± 0.13 ^b,B^	7.07 ± 1.03 ^ab,A^
	ChCl:LA	0.36 ± 0.02 ^a,C^	1.03 ± 0.06 ^c,B^	4.61 ± 0.23 ^cd,A^
	ChCl:MA	<LOQ	0.98 ± 0.08 ^c^	3.57 ± 0.28 ^de^
	ChCl:Xyl	<LOQ	0.23 ± 0.01 ^d^	6.72 ± 0.04 ^ab^
	Ethanol	<LOQ	0.13 ± 0.005 ^d^	5.76 ± 0.22 ^bc^

ChCl:CA: ChCl:CA (2:1), ChCl:Glu: ChCl:Glu (2:1), ChCl:Gly: ChCl:Gly (1:2), ChCl:LA: ChCl:LA (1:1), ChCl:MA: ChCl:MA (2:1), ChCl:Xyl: ChCl:Xyl (1:1). Results are expressed as mean ± standard deviation. Different lower-case letters within the columns and upper-case letters within the rows represent statistically significant differences (*p* < 0.05). LOQ: limit of quantification.

**Table 9:** Flavonoids in QC extracts during in vitro digestion by solvent (µg g^−1^).

Flavonoids	Solvent	Undigested	Gastric Digestion	Intestinal Digestion
Epicatechin	ChCl:CA	<LOQ	0.18 ± 0.004 ^b^	0.51 ± 0.03 ^c^
	ChCl:Glu	<LOQ	0.60 ± 0.01 ^a^	2.37 ± 0.04 ^a^
	ChCl:Gly	<LOQ	0.53 ± 0.05 ^b^	2.38 ± 0.13 ^a^
	ChCl:LA	<LOQ	0.10 ± 0.004 ^c^	0.56 ± 0.03 ^c^
	ChCl:MA	<LOQ	0.05 ± 0.002 ^c^	0.22 ± 0.02 ^d^
	ChCl:Xyl	<LOQ	0.04 ± 0.005 ^c^	1.61 ± 0.02 ^b^
	Ethanol	<LOQ	<LOQ	1.42 ± 0.07 ^b^
Rutin	ChCl:CA	0.12 ± 0.002 ^a,C^	0.18 ± 0.004 ^a,B^	0.60 ± 0.01 ^a,A^
	ChCl:Glu	0.09 ± 0.01 ^b,C^	0.18 ± 0.007 ^a,B^	0.61 ± 0.03 ^a,A^
	ChCl:Gly	0.06 ± 0.004 ^c,B^	0.13 ± 0.004 ^bc,B^	0.45 ± 0.15 ^ab,A^
	ChCl:LA	0.11 ± 0.006 ^ab,C^	0.15 ± 0.008 ^b,B^	0.42 ± 0.001 ^ab,A^
	ChCl:MA	<LOQ	0.11 ± 0.008 ^cd^	0.34 ± 0.04 ^b^
	ChCl:Xyl	<LOQ	0.07 ± 0.008 ^e^	0.49 ± 0.01 ^ab^
	Ethanol	<LOQ	0.09 ± 0.002 ^de^	0.37 ± 0.02 ^ab^
Isoquercitrin	ChCl:CA	<LOQ	0.07 ± 0.004 ^a^	0.25 ± 0.01 ^a^
	ChCl:Glu	0.02 ± 0.004 ^b,B^	0.07 ± 0.007 ^a,B^	0.26 ± 0.02 ^a,A^
	ChCl:Gly	0.01 ± 0.001 ^b,B^	0.06 ± 0.004 ^a,B^	0.21 ± 0.06 ^ab,A^
	ChCl:LA	0.03 ± 0.003 ^a,C^	0.06 ± 0.003 ^a,B^	0.15 ± 0.004 ^ab,A^
	ChCl:MA	<LOQ	0.04 ± 0.002 ^b^	0.12 ± 0.03 ^b^
	ChCl:Xyl	<LOQ	0.02 ± 0.002 ^b^	0.17 ± 0.02 ^ab^
	Ethanol	<LOQ	0.03 ± 0.001 ^b^	0.15 ± 0.004 ^ab^
Kaempferol	ChCl:CA	<LOQ	<LOQ	<LOQ
	ChCl:Glu	<LOQ	<LOQ	<LOQ
	ChCl:Gly	<LOQ	<LOQ	<LOQ
	ChCl:LA	<LOQ	<LOQ	<LOQ
	ChCl:MA	<LOQ	<LOQ	<LOQ
	ChCl:Xyl	<LOQ	<LOQ	<LOQ
	Ethanol	<LOQ	<LOQ	<LOQ
Quercetin	ChCl:CA	<LOQ	0.16 ± 0.008	0.45 ± 0.008 ^b^
	ChCl:Glu	<LOQ	<LOQ	<LOQ
	ChCl:Gly	<LOQ	<LOQ	<LOQ
	ChCl:LA	<LOQ	0.11 ± 0.01	0.02 ± 0.001 ^c^
	ChCl:MA	<LOQ	<LOQ	0.77 ± 0.03 ^a^
	ChCl:Xyl	<LOQ	<LOQ	<LOQ
	Ethanol	<LOQ	<LOQ	<LOQ
∑Flavonoids	ChCl:CA	0.12 ± 0.002 ^a,C^	0.59 ± 0.004 ^c,B^	1.80 ± 0.07 ^bc,A^
	ChCl:Glu	0.11 ± 0.02 ^ab,C^	0.86 ± 0.03 ^a,B^	3.24 ± 0.02 ^a,A^
	ChCl:Gly	0.07 ± 0.006 ^b,B^	0.72 ± 0.04 ^b,B^	3.03 ± 0.35 ^a,A^
	ChCl:LA	0.14 ± 0.008 ^a,B^	0.31 ± 0.008 ^d,B^	1.15 ± 0.04 ^d,A^
	ChCl:MA	N/A	0.30 ± 0.02 ^d^	1.46 ± 0.12 ^cd^
	ChCl:Xyl	N/A	0.13 ± 0.02 ^e^	2.27 ± 0.05 ^b^
	Ethanol	N/A	0.12 ± 0.003 ^e^	1.94 ± 0.08 ^bc^

ChCl:CA: ChCl:CA (2:1), ChCl:Glu: ChCl:Glu (2:1), ChCl:Gly: ChCl:Gly (1:2), ChCl:LA: ChCl:LA (1:1), ChCl:MA: ChCl:MA (2:1), ChCl:Xyl: ChCl:Xyl (1:1). Results are expressed as mean ± standard deviation. Different lower-case letters within the columns and upper-case letters within the rows represent statistically significant differences (*p* < 0.05). LOQ: limit of quantification.

## Data Availability

The original contributions presented in this study are included in the article/Supplementary Materials. Further inquiries can be directed to the corresponding author.

## Supplementary Materials

The following supporting information can be downloaded at: https://www.mdpi.com/article/10.3390/foods14203507/s1, Figure S1: Representative FTIR spectra highlighting O-H stretch and carbonyl regions; Figure S2: Representative LC–MS chromatograms of QP extracts; Figure S3: Representative LC–MS chromatograms of QC extracts; Table S1: Gradient elution used for phenolic compound determination by LC-MS/MS.
