# Supplementary material for: Valorization of Quince By-Products Using Natural Deep Eutectic Solvents (NADES): Extraction and In Vitro Digestion of Phenolic Compounds
Source: Foods. 2025 Oct 15;14(20):3507. doi: 10.3390/foods14203507 (PMC12564506; doi:10.3390/foods14203507)
Supplement: Supplementary file 1 [file foods-14-03507-s001.zip › foods-3900775-supplementary.pdf]

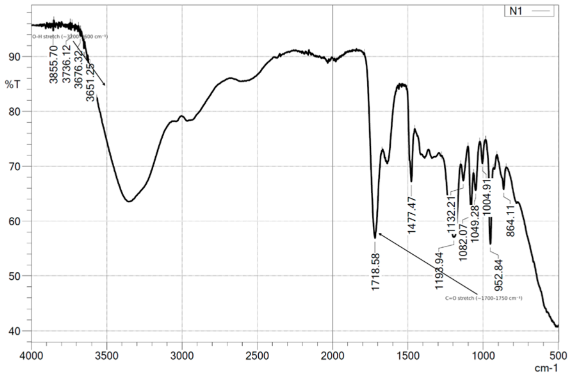
N1
%T
O-H stretch (~3200–3600 cm⁻¹)
3855.70
3736.12
3676.32
3651.23
1718.58
1477.47
1193.94
1132.21
1082.07
1049.28
1004.91
952.84
864.11
C=O stretch (~1700–1750 cm⁻¹)
cm-1

(a)

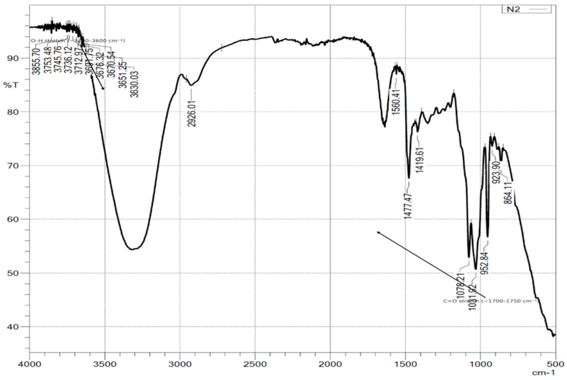
N2
%T
2926.01
1560.41
1419.61
1477.47
923.90
864.11
952.84
C=O stretch (~1700–1750 cm⁻¹)
cm-1

(b)

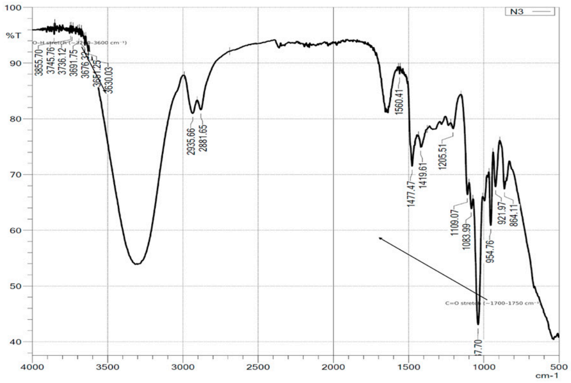
N3
%T
2935.66
2881.65
1560.41
1477.47
1419.61
1205.51
1109.07
1083.99
954.76
921.97
864.11
C=O stretch (~1700–1750 cm⁻¹)
cm-1

(c)

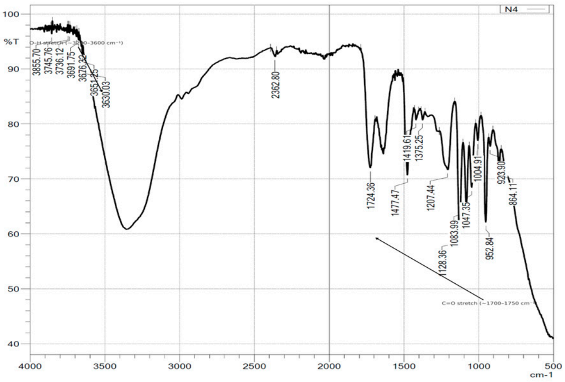
N4
%T
2362.80
1724.36
1477.47
1419.61
1375.25
1207.44
1083.99
1047.35
1004.91
923.90
864.11
952.84
C=O stretch (~1700–1750 cm⁻¹)
cm-1

(d)

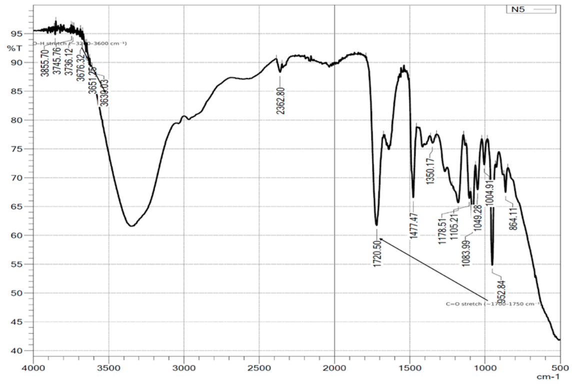
N5
%T
2362.80
1720.50
1477.47
1350.17
1178.51
1105.21
1083.99
1049.28
1004.91
864.11
952.84
C=O stretch (~1700–1750 cm⁻¹)
cm-1

(e)

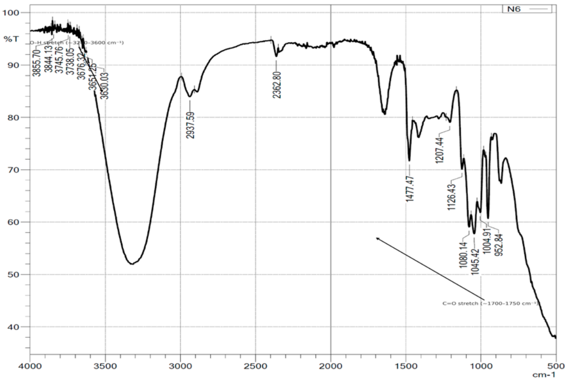
N6
%T
2937.59
2362.80
1477.47
1207.44
1126.43
1080.14
1045.42
1004.91
952.84
C=O stretch (~1700–1750 cm⁻¹)
cm-1

(f)

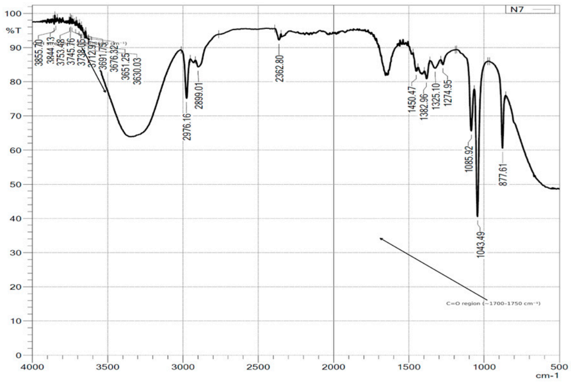
N7
%T
2976.16
2899.01
2362.80
1450.47
1382.96
1325.10
1274.95
1085.92
1043.49
877.61
C=O region (~1700–1750 cm⁻¹)
cm-1

(g)

Figure S1. Representative FTIR spectra highlighting O-H stretch and carbonyl regions. (a) Choline chloride:citric acid (2:1), (b) Choline chloride:glucose (2:1), (c) Choline chloride:glycerol (1:2), (d) Choline chloride:lactic acid (1:1), (e) Choline chloride:malic acid (2:1), (f) Choline chloride:xylitol (1:1), (g) Ethanol (70 %)

Key spectral regions/peaks are marked with arrows to aid interpretation and highlight solvent-specific patterns.

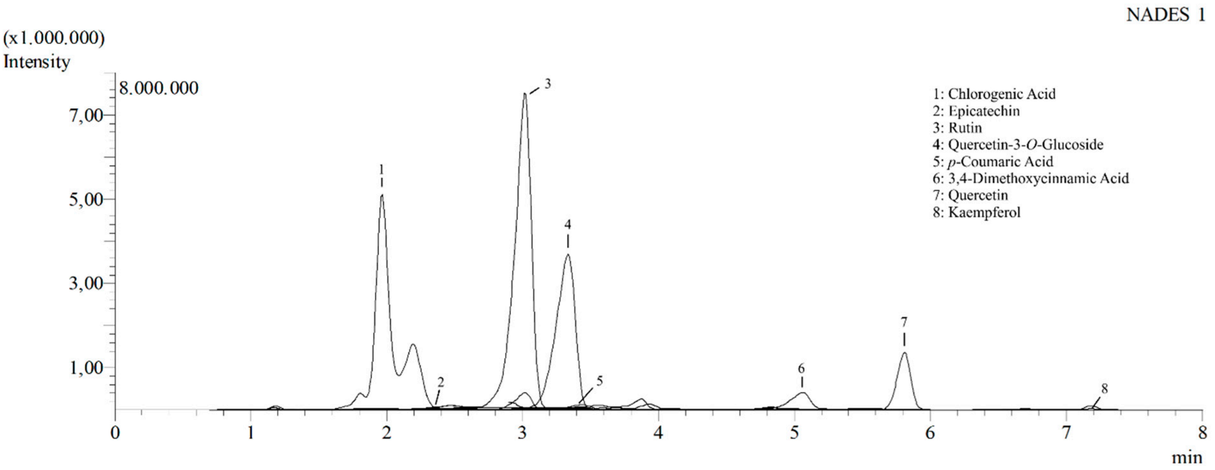

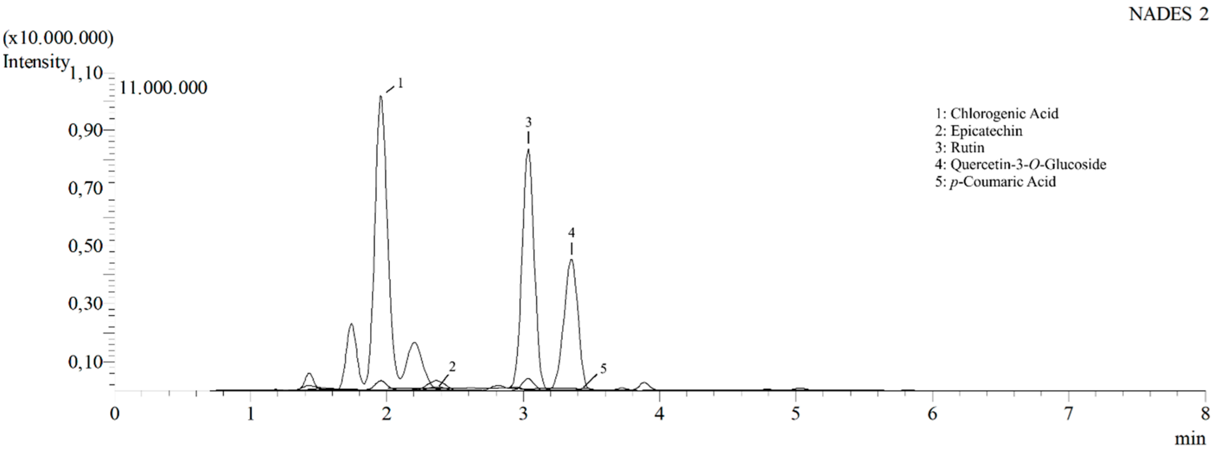

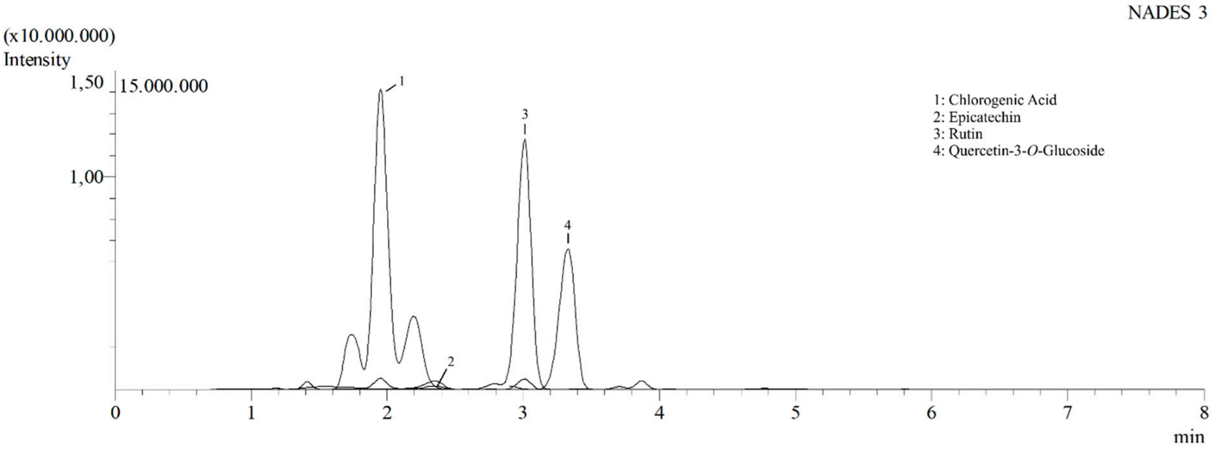

NADES 4

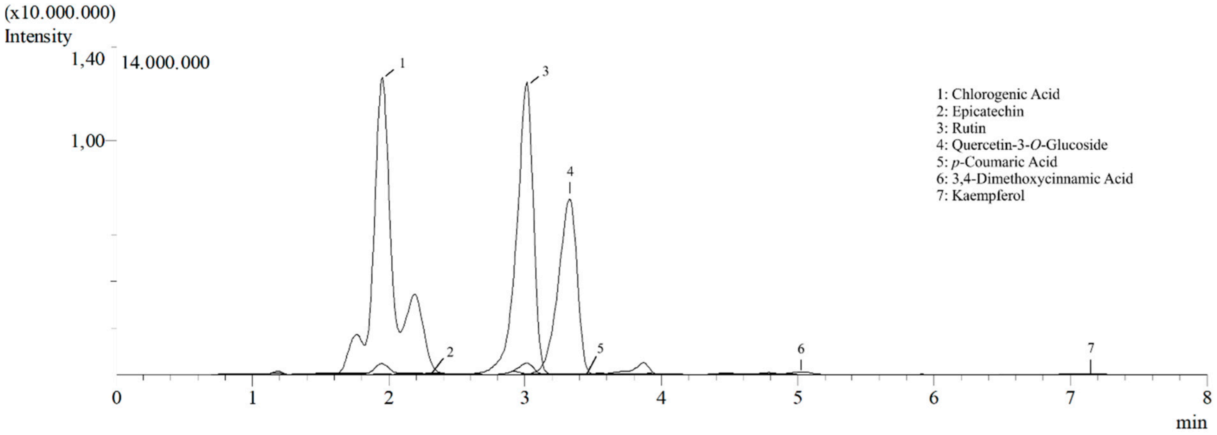

NADES 5

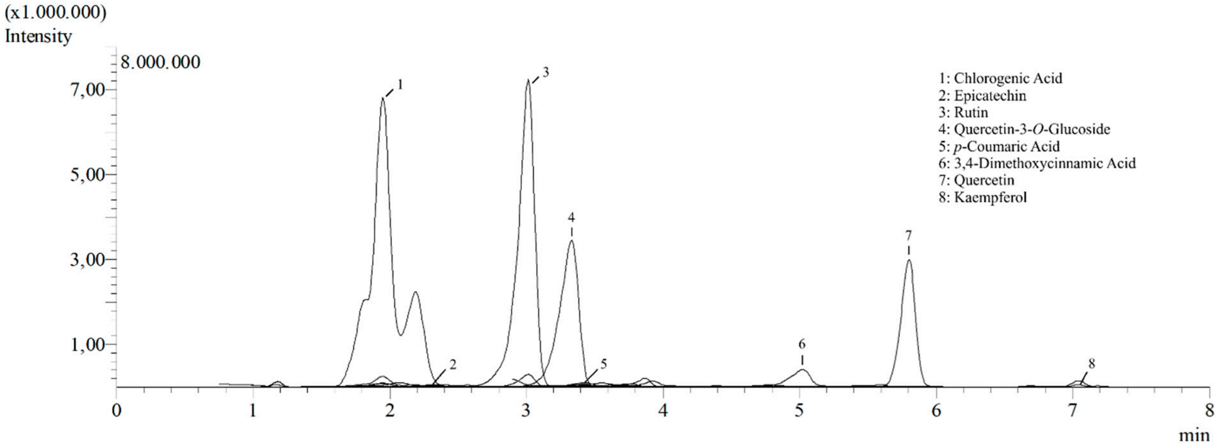

NADES 6

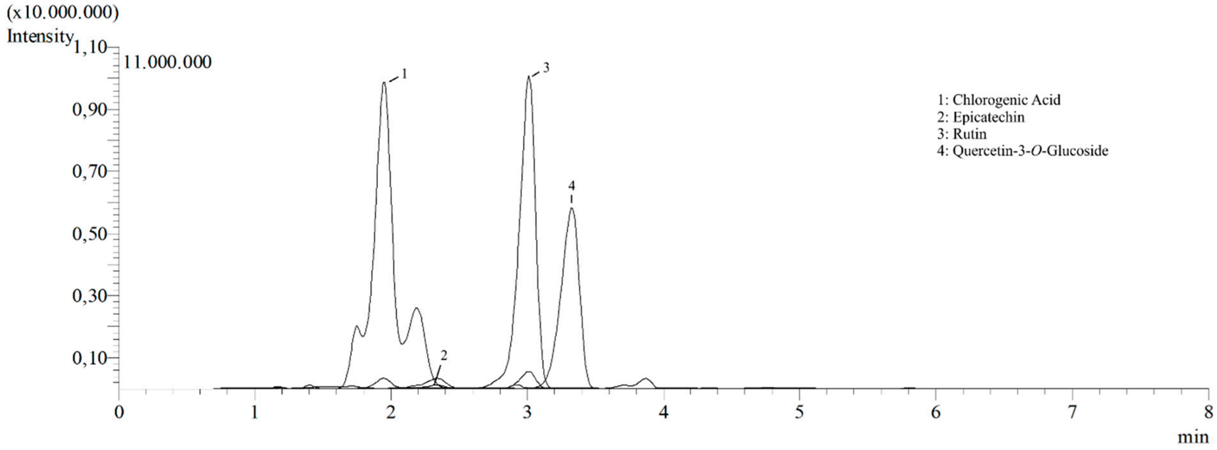

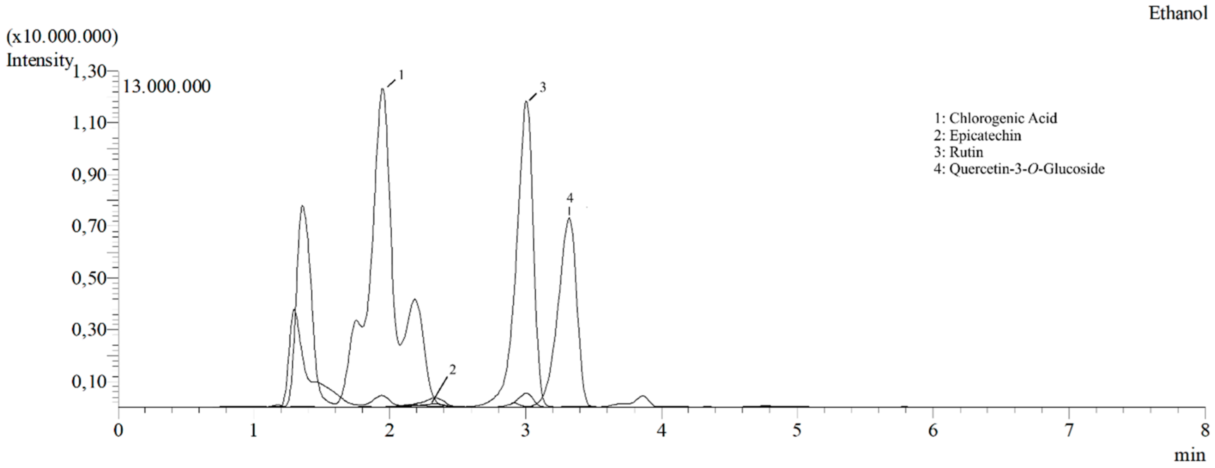

Figure S2. Representative LC–MS chromatograms of QP extracts. Compounds <LOQ are not marked.

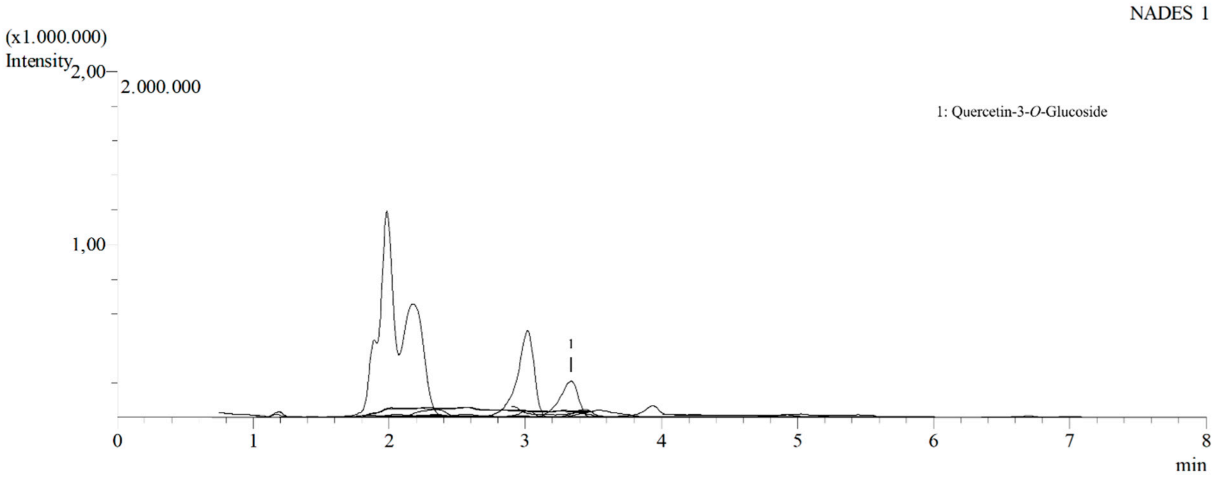

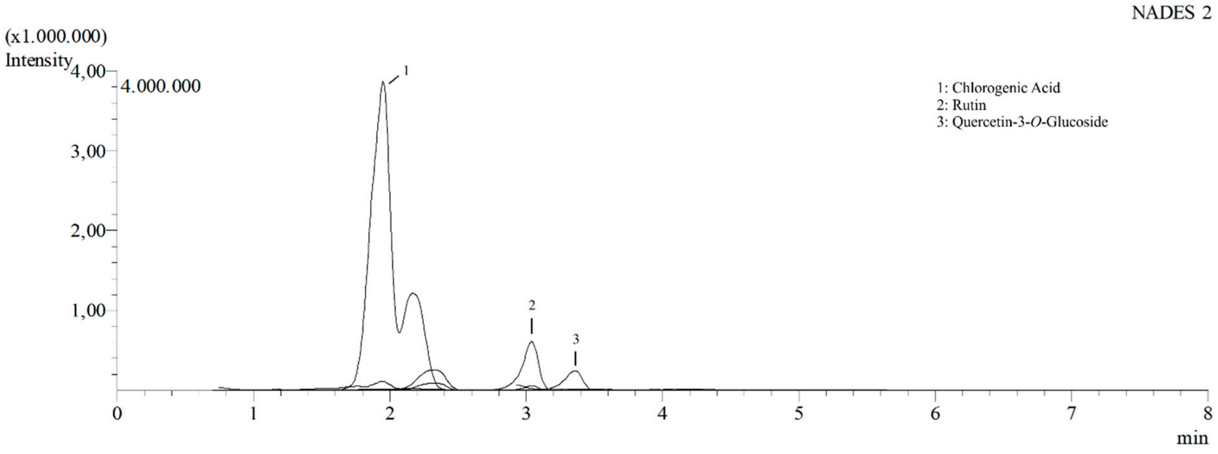

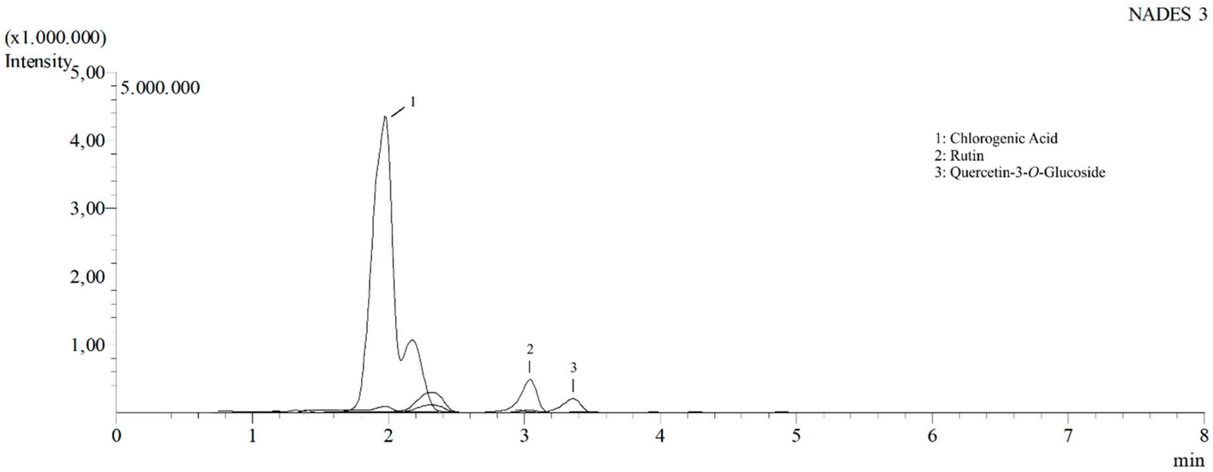
NADES 3
(x1.000.000)
Intensity
5,00
5.000.000
4,00
3,00
2,00
1,00
1
2
3
1: Chlorogenic Acid
2: Rutin
3: Quercetin-3-O-Glucoside
0
1
2
3
4
5
6
7
8
min

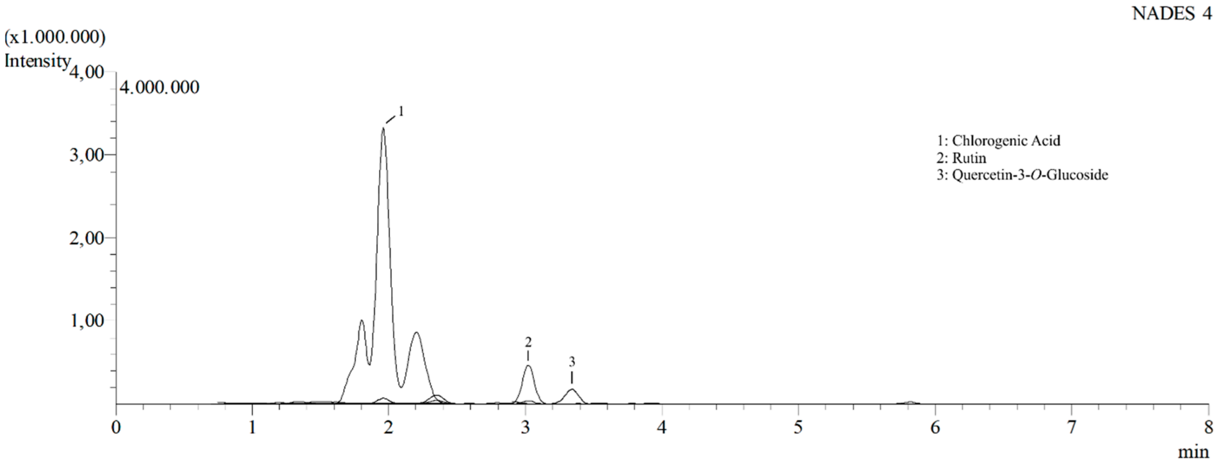
NADES 4
(x1.000.000)
Intensity
4,00
4.000.000
3,00
2,00
1,00
1
2
3
1: Chlorogenic Acid
2: Rutin
3: Quercetin-3-O-Glucoside
0
1
2
3
4
5
6
7
8
min

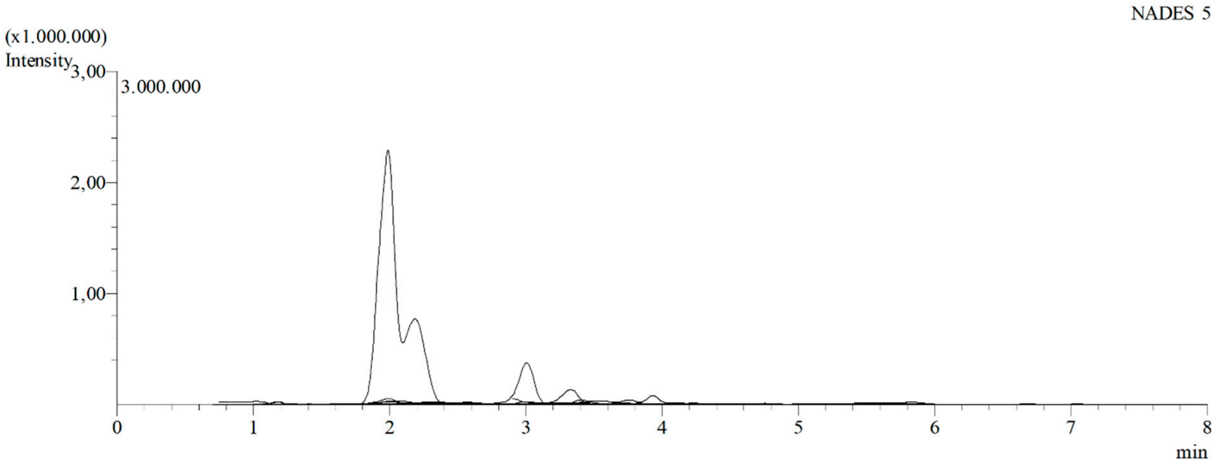
NADES 5
(x1.000.000)
Intensity
3,00
3.000.000
2,00
1,00
0
1
2
3
4
5
6
7
8
min

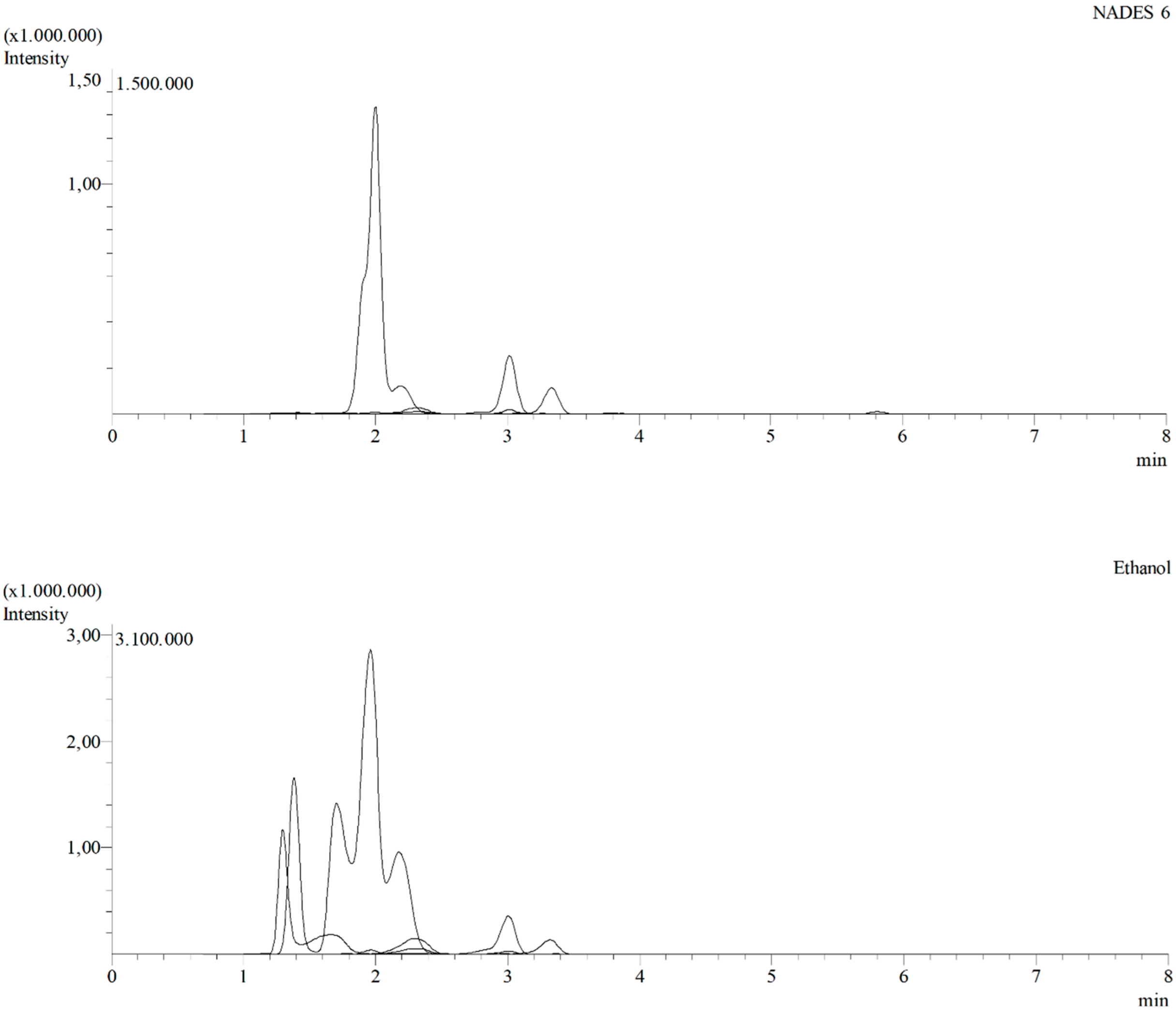

Figure S3. Representative LC–MS chromatograms of QC extracts. Compounds <LOQ are not marked.

Table S1. Gradient elution used for phenolic compound determination by LC-MS/MS

Time (min)	Eluent A (%)	Eluent B (%)
0.00	80	20
0.50	80	20
7.00	50	50
12.00	5	95
12.10	80	20
15.00	80	20

A: Ultrapure water, B: Acetonitrile
